# Amine Exchange of Aminoalkylated Phenols as Dynamic Reaction in Benzoxazine/Amine‐Based Vitrimers

**DOI:** 10.1002/marc.202400557

**Published:** 2024-10-10

**Authors:** Adrian Wolf, Lea Pursche, Laura Boskamp, Katharina Koschek

**Affiliations:** ^1^ Fraunhofer Institute for Manufacturing Technology and Advanced Materials (IFAM) Wiener Straße 12 28359 Bremen Germany; ^2^ Department 2 Biology/Chemistry University of Bremen Leobener Straße 3 28359 Bremen Germany; ^3^ Faculty of Production Engineering University of Bremen Bibliothekstraße 1 28359 Bremen Germany

**Keywords:** amine, aminoalkylated phenol, benzoxazine, reaction mechanism, vitrimer

## Abstract

Bisfunctional benzoxazine and polyether diamine‐based polymers show Arrhenius‐like stress‐relaxation varying with stoichiometry and polymerization temperatures proving vitrimeric behavior. Molecular structural investigations reveal the presence of different aminoalkylated phenols occurring at varying ratios depending on polymer composition and polymerization conditions. The vitrimeric mechanism is found to involve an amine exchange reaction of aminoalkylated phenols in an equilibrium reaction like a nucleophilic substitution reaction. As determined by molecular studies and dissolution experiments in reactive solvents, aliphatic and aromatic primary as well as aliphatic secondary amines in the polybenzoxazine structure can act as nucleophiles in reaction with electrophilic methylene bridges. Thus, aminoalkylated phenols proved to be a relevant structural motif resulting in a vitrimeric polybenzoxazine due to amine exchange reaction.

## Graphical abstract

Benzoxazine/amine‐based vitrimers comprise complex crosslinked aminoalkylated phenolic structures depending on stoichiometries and polymerization conditions. They can undergo nucleophilic substitution‐like (S_N_‐like) amine exchange equilibrium reactions involving nucleophilic primary and secondary amines and electrophilic methylene bridges, enabling vitrimer behavior.

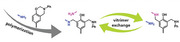

## 1 Introduction

The consumption of polymeric materials and the associated waste generation more than doubled since the millennium. Yet, low recycling rates and thus consumption of non‐renewable resources remain major challenges.^[^ ^1^ ^]^ Especially regarding structural applications with high requirements in strength and toughness, vitrimers have emerged as a new and potentially more sustainable alternative.^[^ ^2^ ^]^ Vitrimers comprise both, the high mechanical properties and chemical resistance of thermosets, as well as the reprocessability and recyclability^[^ ^3^ ^]^ of thermoplastics.^[^ ^4^ ^]^ The thermoset‐like properties, expressed at low temperatures, arise from a rigid, covalently crosslinked polymer structure.^[^ ^5^ ^]^ At elevated temperatures, the crosslinks can undergo reversible dynamic exchange reactions on the molecular level, which, on the macroscopic level allow the materials to flow, express Arrhenius‐like stress‐relaxation behaviour,^[^ ^6^ ^]^ and often show solubility in specific reactive solvents.^[^ ^7^ ^]^ Manifold characteristic functional groups have been described to enable vitrimeric behaviour. Among others, prominent examples exploited chemical reactions like transesterification,^[^ ^8^, ^9^, ^10^ ^]^ disulfide metathesis,^[^ ^11^, ^12^ ^]^ diselenide metathesis,^[^ ^13^ ^]^ transamination of vinylogous urethanes,^[^ ^14^ ^]^ or dioxaborolane metathesis.^[^ ^15^ ^]^ Aside resin systems like epoxy or polyurethane, polybenzoxazines that have high molecular design flexibility and superior properties like mechanical strength, near‐zero shrinkage upon polymerization, and intrinsic flame retardance^[^ ^16^ ^]^ have become a promising starting point for the formation of vitrimers. The most promising polybenzoxazine‐based vitrimers are based on chemical reactions such as internally catalyzed transesterification, imine metathesis, transcarbamoylation, or transalkylation of trialkylsulfonium salts among others.^[^ ^17^, ^18^, ^19^, ^20^, ^21^ ^]^

Benzoxazines (BZ) undergo thermally induced ring opening polymerization (ROP), resulting in highly crosslinked polymers. *para*‐substituted BZ result in phenoxy type poly(BZ) structures that form at lower polymerization temperatures and rearrange into phenolic type structures at elevated temperatures.^[^ ^16^ ^]^ The ROP does not require catalysts, however, the resulting acidic hydroxyl group can have an autocatalytic effect. Aside acid or base catalysis, BZ are also prone to react with strong nucleophiles, e.g. amines or thiols.^[^ ^16^, ^22^, ^23^ ^]^ Recently, amines have been extensively studied as co‐reactants for BZ, aiming at lowering the polymerization temperature and increasing material toughness. The BZ/amine reaction mechanism was described to proceed via reversible formation of ring‐opened BZ‐amine addition‐product intermediates^[^ ^24^, ^25^, ^26^ ^]^ that can act as latent curing systems^[^ ^27^ ^]^ but overall BZ/amine reaction is irreversible.^[^ ^28^ ^]^

Recently, we have proven that a bisphenol‐A and aniline‐based BZ (BA‐a) and the polyetheramine ED600 result in a dynamic polymer network showing vitrimeric characteristics.^[^ ^29^ ^]^ Aside thermoset‐like mechanical properties at low and operating temperatures, the three‐dimensionally crosslinked polymer networks exhibit Arrhenius‐like stress‐relaxation behavior and reprocessability at elevated temperatures. That points to covalent exchange reactions in a polybenzoxazine/amine network lacking any vitrimeric functional group described above, e.g. esters, disulfides, begging the question of the responsible structural motif and reaction mechanism behind the vitrimeric properties.^[^ ^29^ ^]^

This contribution derives the dynamic reaction mechanism of this novel polybenzoxazine vitrimer investigating the responsible structural motifs, the impact of monomer composition as well as polymerization conditions.

## 2 Results and Discussion

### 2.1 Vitrimeric Behavior of BZ/Amine Thermosets

Aiming at a deeper understanding of BZ/amine thermosetting structure relating to dynamic polymer network properties,13 BA‐a and polyether diamine ED600 formulations were prepared with varying in BZ/amine ratios (1:1, 1:0.5, 1:0.25, 1:0) and polymerization conditions (a) 120 °C; b) 120, 150 °C; c) 120, 150, and 180 °C or d) (only for neat BZ according to manufacturer protocol) 180, 200 °C; 2 h respectively) (Infrared spectra (IR) in Figure S1, Supporting Information). Six representative samples were used in stress relaxation measurements and the normalized relaxation moduli G at different temperatures were recorded as a function of time and the respective relaxation times τ (i.e., the times by which G/G^0^ was reduced to 1/e) were fitted according to linearized Arrhenius‐equation (Equation (1)).

(1) $$\ln\left(\tau \left(T\right)\right)=\;\ln\tau^{0}+\frac{E_{a}}{R}\cdot \frac{1}{T}$$

**Figure 1:**
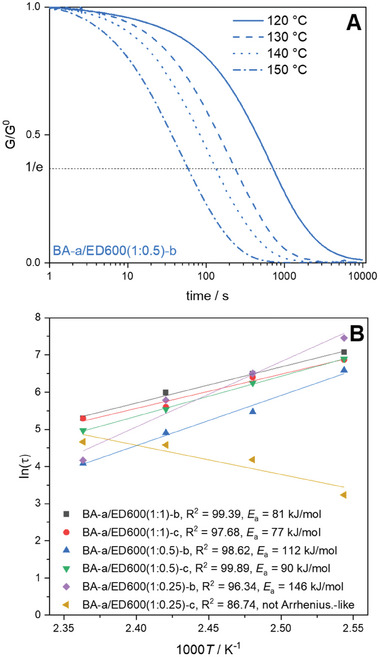
A) Normalized relaxation moduli, exemplary shown for polymer sample BA‐a/ED600(1:0.5)‐b at different temperatures. B) Fitting of the relaxation times according to Arrhenius Equation (1).

Arrhenius‐like temperature dependency of the relaxation times is indicative for the presence of vitrimers and can be explained by the fact that the rate of stress‐relaxation is limited by the rate of the exchange reaction. The slope of the fitted plots was used to calculate the respective activation energies of the exchange reaction *E*_a_ for the different polymer compositions and polymerization conditions (**Figure** **1**; Figure S2–S6, Supporting Information). Non‐normalized stress‐relaxation curves are shown in Figure S7, Supporting Information).

Except for BA‐a/ED600(1:0.25)‐c, all samples show quick stress relaxation according to Arrhenius equation (Equation (1) and Figure 1B) with activation energies increasing with decreasing amine content. The sample BA‐a/ED600(1:0.25)‐c does not show stress relaxation proving that the presence of amines is essential. The resulting polyBZ/polyetheramine polymer networks feature structural motifs allowing dynamic covalent exchange reactions. Furthermore, polymerization temperature plays a role as activation energies increase with increasing temperature. Deviating activation energies indicate either changes in the reactants involved in the exchange reactions or in their catalytic reactivity. In case of low amine content the high polymerization temperature in case of non‐vitrimeric BA‐a/ED600(1:0.25)‐c results in a molecular structure similar to the conventional poly(BZ), which is known to be non‐dynamic.^[^ ^29^ ^]^

Since the BZ/amine thermosets lack any functional group known to enable vitrimer behavior a novel vitrimeric reaction is assumed to occur arising from the incorporation of amines into polyBZ network structure.

### 2.2 Polymerization and Structure Elucidation of Benzoxazine/Amine Polymers

For the investigation of the reaction mechanism resulting in vitrimeric BZ/amine polymer structures, the monofunctional *para*‐cresol and aniline‐based benzoxazine (C‐a) was chosen as model system due to its similar chemical reactivity to the BZ in the BA‐a and ED600 system and used in reaction with different amines, including primary polyether monoamine M600NH_2_ as well as further primary, secondary and tertiary amines, varying in nucleophilicity and basicity.

**Scheme 1:**
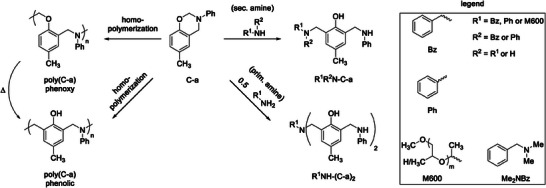
Molecular structures resulting from *para*‐cresol and aniline‐based BZ (C‐a) homopolymerization and BZ/amine reaction of C‐a with different amines.

While the ROP of neat C‐a results solely in phenolic type poly(C‐a) structures even at lower polymerization temperatures, expressing tertiary amine structures and C^ar^CH_2_NR_2_ methylene bridges, the reaction with additional primary or secondary amines results in a variety of *ortho*‐aminoalkylated phenols (**Scheme** **1**). Equimolar reactions with primary amines yield secondary amine structures with C^ar^CH_2_NHR methylene bridges, and reactions with 0.5 equivalents of primary or 1 equivalent of secondary amine yield tertiary amine structures with C^ar^CH_2_NR_2_ methylene bridges. For all reactions of C‐a with primary amines, the reaction also yields the other aminoalkylated phenol structure in smaller amounts and all C‐a/amine reactions yield minor amounts of poly(C‐a).

#### 2.2.1 Dependence of Stoichiometry and Polymerization Temperature on BZ/Amine Reaction

The bulk reaction of C‐a and M600NH_2_ (R^1^NH_2_ with R^1^ = M600) at varying ratios (C‐a/M600NH_2_ = 1:1, 1:0.5, 1:0.25) conducted at 120 °C for 2 h yields mixtures containing M600NH‐C‐a, M600N‐(C‐a)_2_ and unreacted C‐a (**Table** **1**, Table S1 and Figure S8, Supporting Information).

**Table 1:** Product ratio (yields from ^1^H‐NMR, Figure S8 and Table S1, Supporting Information) of C‐a/M600NH_2_ (1:1, 1:0.5, 1:0.25, 1:0) bulk reactions after 120 min at 120 °C and respective reaction enthalpies from DSC associated with formation of poly(C‐a) (ΔH_ROP_) and BZ/amine products (ΔH_BZ/amine_).

molar ratio C‐a/M600	M600N‐(C‐a)_2_/M600NH‐C‐a	ΔH_BZ/amine_ / J/g	ΔH_ROP_ / J/g
1:1	0.22	33	21
1:0.5	0.39	31	77
1:0.25	0.61	16	142
1:0	–	–	171

The equimolar reaction of BZ and polyetheramine (C‐a/M600NH_2_  =  1:1) results in monosubstituted polyetheramine M600NH‐C‐a as the main reaction product and a minor content of disubstituted amine yielding M600N‐(C‐a)_2_ with a ratio of 0.22. The content of M600N‐(C‐a)_2_ increases gradually to 0.39 and 0.61 for decreasing amine contents C‐a/M600NH_2_ of 1:0.5 and 1:0.25, respectively. At low amine content the remaining excess of C‐a can undergo the second reaction step with the initially formed M600NH‐C‐a reaction product thus increasing the formation of the tertiary amine M600N‐(C‐a)_2_.^[^ ^25^ ^]^

After 2 h at 120 °C, the equimolar C‐a/M600NH_2_ reaction yields 16% of unreacted C‐a. Decrease in the amine content leads to an increase in the yield of unreacted C‐a (up to 47 % for C‐a/M600NH_2_ ratio of 1:0.25). Since each primary amino group can react with a maximum of two oxazine rings, only ≈ 50% of the C‐a can react with M600NH_2_ in a 1:0.25 mixture.

**Figure 2:**
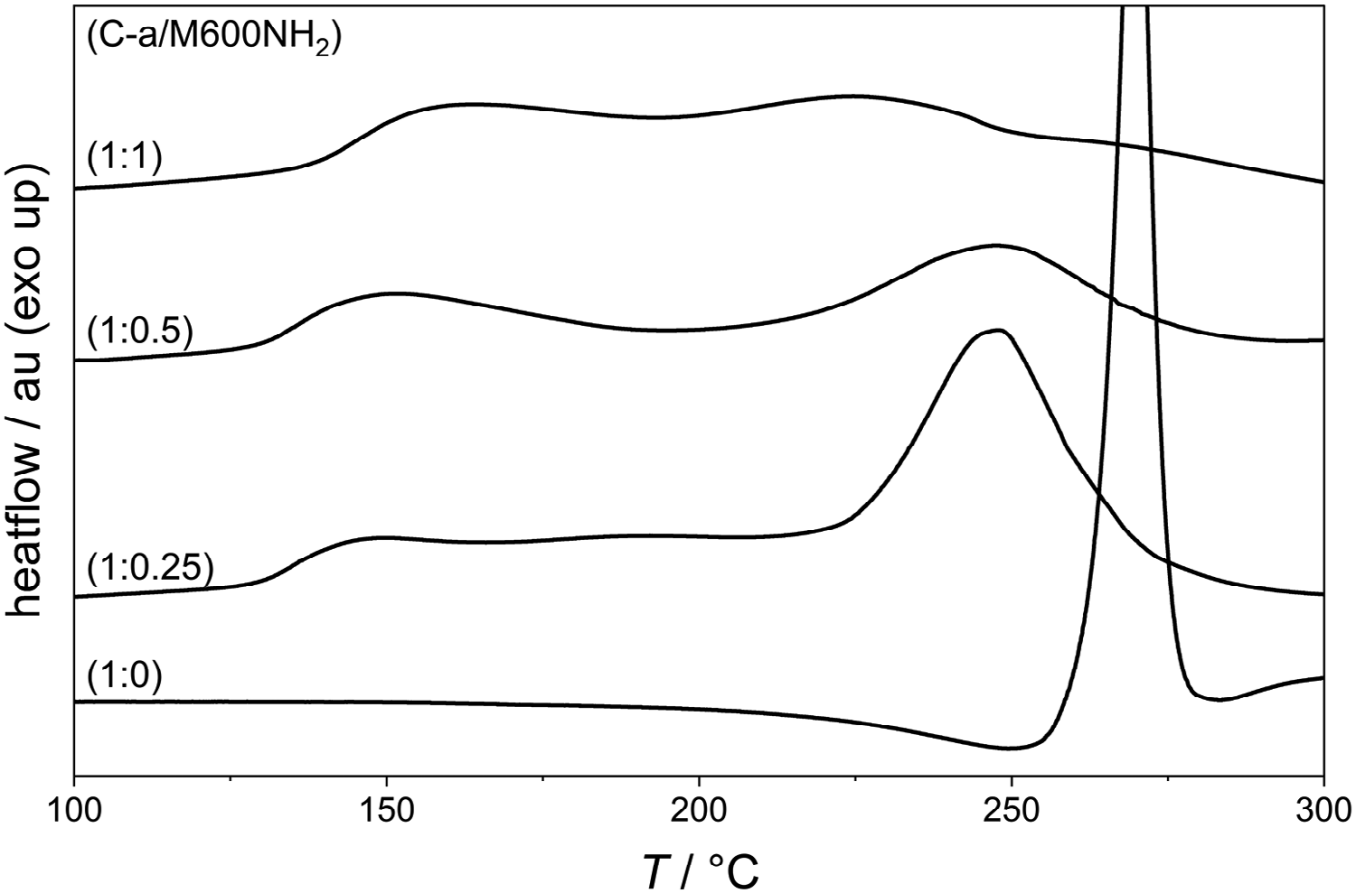
DSC thermograms of bulk reactions of C‐a and M600NH_2_ at different stoichiometries.

The unreacted C‐a can undergo homopolymerization at elevated temperatures. To investigate this, differential scanning calorimetry (DSC) measurements were performed for stoichiometric ratios of C‐a/M600NH_2_ of 1:1, 1:0.5, 1:0.25, and 1:0 (neat C‐a) (Table 1, **Figure** **2**).

The neat C‐a sample shows a single exothermic signal at *T*_onset_  =  264 °C corresponding to the ROP known for BZ. All amine containing C‐a mixtures show two exothermic signals – a low temperature signal (*T*_onset_  =  131–139 °C) most likely corresponding to the BZ/amine reaction, and a high temperature signal (*T*_onset_  =  215–224 °C) that can be aligned to the ROP of the remaining C‐a. Compared to the neat C‐a, the presence of amines lowers the ROP *T*_onset_, which can probably be explained by catalytic effects for example by acidic phenolic protons of the M600NH‐C‐a and M600N‐(C‐a)_2_ products or amines. The reaction enthalpy of the C‐a ring opening event increases with increasing C‐a content, while the enthalpy associated with the BZ/amine reaction decreases (Δ*H*_ROP_ / J/g =  21 and 142; Δ*H*_BZ/amine_ / J/g  =  33 and 16 for 1 eq. and 0.25 eq. of M600NH_2_, respectively).

In the reaction of bisfunctional BZ with diamine, the formation of amine‐BZ structures (analogous to R^1^R^2^N‐C‐a) corresponds to the formation of linear polymer chains, while the subsequent formation of amine‐BZ_2_ (analogous to R^1^N‐(C‐a)_2_) and poly(BZ) corresponds to the formation of crosslinks. In summary, these results indicate that in BZ/amine polymerization, lower amine contents lead to an increased formation of amine‐BZ_2_ and poly(BZ) crosslinks in the polymer, provided that sufficiently high reaction temperatures are applied. Higher amine contents, on the other hand, lead to the increased formation of amine‐BZ structures and thus materials with lower crosslinking density.

#### 2.2.2 Impact of Amine Reactivity on Benzoxazine Ring Opening Polymerization

**Figure 3:**
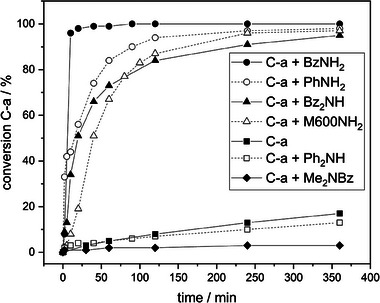
Conversions of neat C‐a or C‐a in equimolar bulk reactions with different amines at 120 °C determined from ^1^H‐NMR spectra (Figures S9–S15, Supporting Information) at time intervals ranging from 2 to 360 min.

ROP of C‐a and monoamines varying in nucleophilicity and basicity was studied at equimolar ratio in bulk reactions at 120 °C with neat C‐a ROP as reference (Scheme 1) (**Figure** **3**).

High C‐a conversion to corresponding C‐a/amine adducts takes place in the presence of nucleophilic amines at high conversion rates according to the order of nucleophilicity (BzNH_2_ > PhNH_2_ ≈ M600NH_2_ ≈ Bz_2_NH). This confirms the results of Sun et al. who reported the nucleophilic attack of the amine at the methylene bridge of the oxazine ring (OCH_2_N) to be the rate determining step in the BZ/amine reaction.^[^ ^28^ ^]^ Ph_2_NH does not induce BZ/amine reaction, due its low nucleophilicity resulting almost exclusively in poly(C‐a) formation at a similar conversion rate to neat C‐a ROP. The aliphatic tertiary amine Me_2_NBz almost fully prevents C‐a conversion due to the absence of active hydrogen atoms. Furthermore, the high basicity reduces autocatalysis from phenolic hydroxyl groups in poly(C‐a) structures yielding a lower conversion rate in comparison to the ROP of neat C‐a.

A similar impact can be expected of the two different secondary amino groups existing in the formed adduct R^1^HN‐C‐a on the C‐a ROP which would result in a crosslinking. Accordingly, the adduct R^1^N‐(C‐a)_2_ lacking active hydrogen in the tertiary amino groups and poly(C‐a) are not expected to induce further BZ/amine reactions.

### 2.3 Dynamic Bond Exchange Mechanism in Benzoxazine/Amine Polymer Networks

**Scheme 2:**
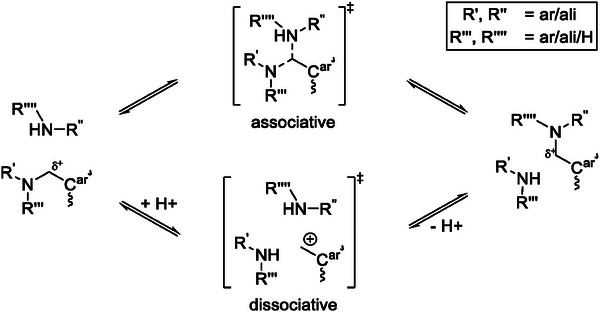
Potential dynamic vitrimer exchange mechanisms in BZ/amine networks. Associative S_N_2‐like (top); dissociative S_N_1‐like (bottom).

The aminoalkylated phenols that occur in BZ/amine‐based polymer networks comprise different characteristic nucleophilic and electrophilic groups that may be involved in dynamic covalent interactions. Potential nucleophiles are both, aromatic and aliphatic primary and secondary amines, as well as tertiary amines and phenolic hydroxyl groups. Potential electrophiles are low field shifted methylene bridges (C^ar^CH_2_NHR or C^ar^CH_2_NR_2_) neighbouring phenolic aromatic rings and amines with various aliphatic or aromatic substituents. A hypothetical exchange mechanism was derived that can explain the nucleophilic substitution reaction (**Scheme** **2**). Vitrimers often rely on associative dynamic exchange reactions, which guarantee the characteristic constant crosslinking density in the polymer. Recently, however, it has been reported that material integrity can also be preserved in case of a dissociative mechanism if the equilibrium constant strongly favors the bonded state.^[^ ^30^, ^31^ ^]^ The associative S_N_2‐like pathway of this mechanism involves attack of a nucleophile at the electrophilic methylene bridge, followed by cleavage of a leaving group, after a proton transfer from the attacking nucleophile. The proton transfer could probably be facilitated by acidic catalysis, e.g from phenolic hydroxyl groups. The dissociative pathway involves S_N_1‐like elimination of the leaving group before addition of the nucleophile. Protonation of the amine before the leaving of the leaving group via acidic catalysis is probably required in this pathway. This allows the leaving group to be a neutral amine rather than the otherwise occurring, highly basic RNH^−^ intermediate. The intermediary primary benzylic carbocation is rather stable due to delocalization of the positive charge in the neighbouring aromatic ring^[^ ^32^ ^]^ and is attacked by nucleophilic amine. Both, S_N_1 and S_N_2‐like mechanisms can explain amine exchange of aminoalkylated phenols and could be followed simultaneously or separately depending on reaction conditions.

#### 2.3.1 Amine Exchange Reactions of Aminoalkylated Phenols in Bulk

**Scheme 3:**
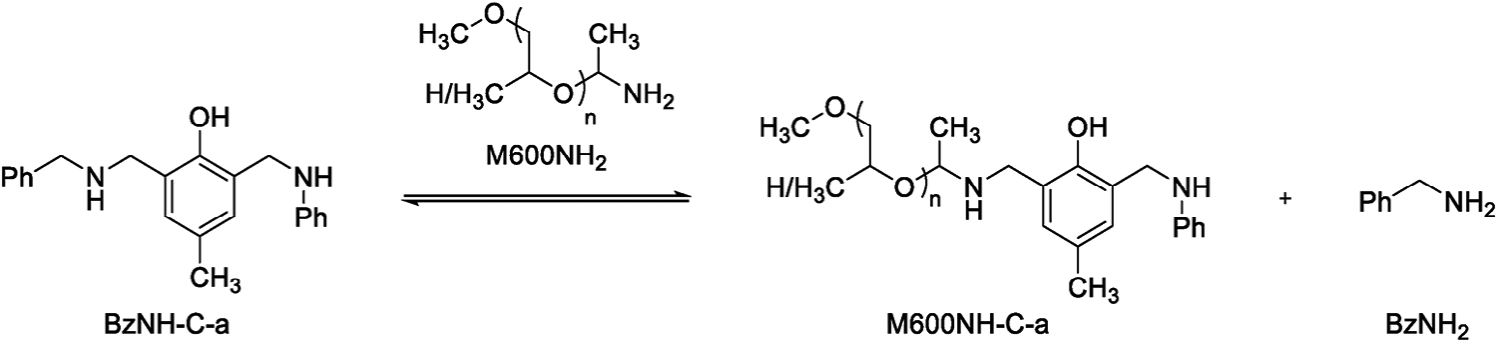
Reaction of BzNH‐C‐a with M600NH_2_ at 120 °C for 24 h.

**Figure 4:**
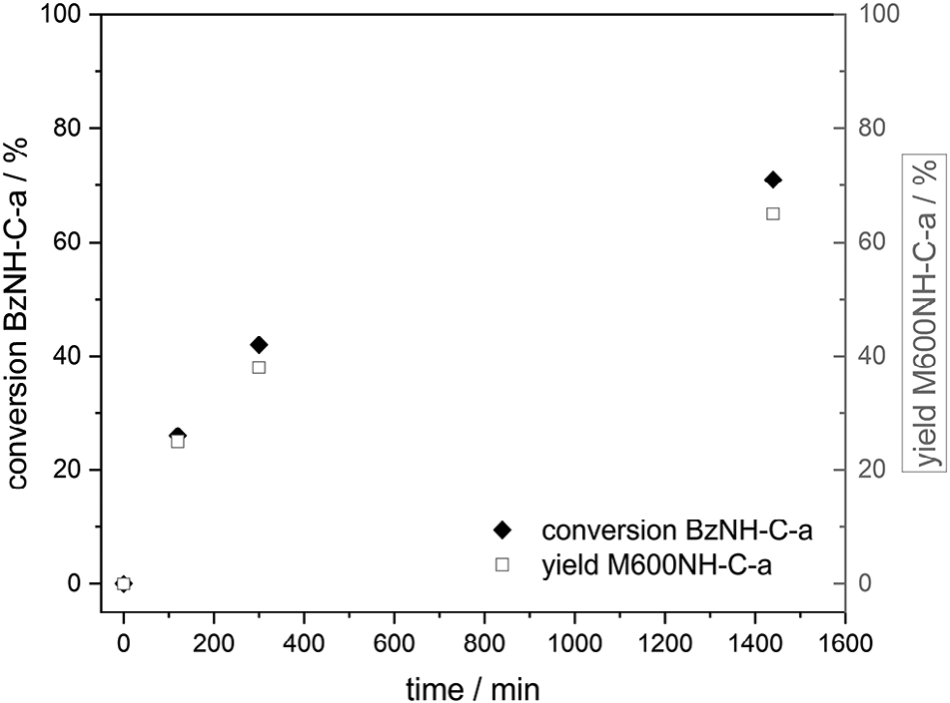
Conversion of BzNH‐C‐a and yield of M600NH‐C‐a in bulk reaction BzNH‐C‐a with M600NH_2_ at 120 °C determined by ^1^H‐NMR at specific intervals.

For a better understanding of the vitrimeric mechanism, the equimolar reaction of pure BzNH‐C‐a and M600NH_2_ was investigated (**Scheme** **3**) and monitored by ^1^H NMR spectroscopy at specific intervals (**Figure** **4**; Figures S16 and S17, Supporting Information).

The resulting product mixture clearly contains M600NH‐C‐a as main reaction product (Figures S16 and S17, Supporting Information). Both, the conversion of BzNH‐C‐a and the yield of M600NH‐C‐a (related to initial BzNH‐C‐a amount) increase with time, approaching saturation and reaching 71 % and 65% after 24 h, respectively. BzNH‐C‐a comprises two different methylene bridges (C^ar^CH_2_NHBz and C^ar^CH_2_NHPh) that could theoretically act as electrophiles in the exchange reaction. The fact that the yield of M600NH‐C‐a almost fully accounts for the converted amount of BzNH‐C‐a, indicates almost exclusive amine exchange reaction of the benzyl bearing methylene bridge (C^ar^CH_2_NHBz), probably due to sterical reasons. The second reaction product BzNH_2_ could not be determined quantitatively due to evaporation. Thermogravimetric analysis coupled with IR (TGA‐IR) measurements of the reaction mixture (Figure S18, Supporting Information) clearly show the formation and evaporation of BzNH_2_ at elevated temperatures. The presence of BzNH_2_ in the reaction mixture was finally proven by NMR spectroscopy investigating the condensate (Figures S16 and S17, Supporting Information). Furthermore, the presence of aniline signals indicated minor amine exchange reaction at the phenyl residue bearing methylene bridge (C^ar^CH_2_NHPh).

These results prove the occurrence of amine exchange reactions of aminoalkylated phenols. The released amine can itself attack present methylene bridges and initiate further amine exchange in an equilibrium reaction, which can explain the characteristic saturation behavior at the observed yields.

#### 2.3.2 Stability and Dissolution of Benzoxazine/Amine Polymer Networks in Solution

**Figure 5:**
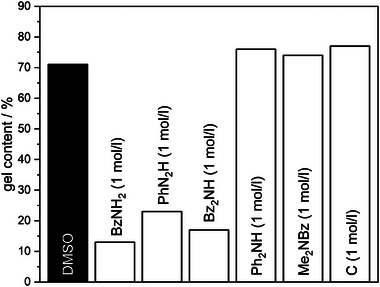
Gel contents of BA‐a/ED600(1:0.5)‐b polymer heated in DMSO or 1 molar solution of different monoamines or monophenol in DMSO (1 mol l^−1^) at 120 °C for 2 h; black column represents dissolution in pure DMSO as reference.

Dissolution rate of the BZ/amine polymer network BA‐a/ED600(1:0.5)‐b was investigated in dimethylsulfoxide (DMSO) solutions in presence of BzNH_2_, Bz_2_NH, PhNH_2_, Ph_2_NH, Me_2_NBz, *para*‐cresol (C) varying in nucleophilicity and the number of active hydrogen atoms. Additional nucleophiles are expected to participate in the dynamic amine exchange reaction yielding chain fragmentation of BZ/amine polymers and by this dissolution. Gel content was determined for all BA‐a/ED600 polymers in amine containing DMSO solutions (Figure S19, Supporting Information). Dissolution experiments were conducted for 2 h at a temperature well above the topology freezing transition temperature (*T*_v_) in order to ensure sufficiently high reaction rates (e.g., BA‐a/ED600(1:0.5)‐b: *T*_v_  =  39 °C^[^ ^29^ ^]^). **Figure** **5** exemplary shows the gel content results for the BA‐a/ED600(1:0.5)‐b.

BA‐a/ED600(1:0.5)‐b treated in pure DMSO does not dissolve and yields a high gel content confirming a high degree of crosslinking. Comparable results were obtained for diphenylamine, cresol, and dimethylbenzylamine, which indicates the low reactivity of these nucleophiles in the exchange reaction. The low reactivity of diphenylamine and cresol can probably be explained by their low nucleophilicity. Dimethylbenzylamine is a stronger nucleophile, however, it lacks active hydrogen atoms. Its low reactivity proves that a proton transfer step is a basic requirement for the dynamic exchange reaction mechanism. The primary amines benzyl‐ and phenylamines, as well as the reactive secondary amine dibenzylamine are strong nucleophiles able to participate in amine exchange reactions and by this increasing dissolution of BZ/amine polymer networks.

**Figure 6:**
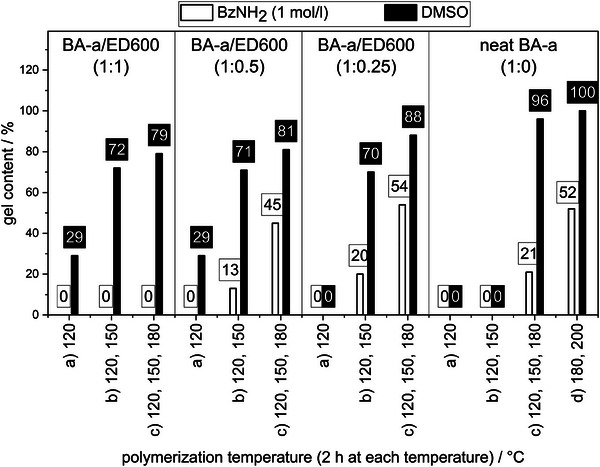
Gel contents of different BA‐a/ED600 polymers or neat poly(BA‐a) polymerized at different conditions heated in of BzNN_2_ in DMSO (1 mol l^−1^) or DMSO at 120 °C for 2 h. All results are depicted in Figure S19 (Supporting Information).

Aside the amine reactivity, dissolution is affected by BZ/amine polymer composition and polymerization temperature as shown for BA‐a/ED600 polymers and neat poly(BA‐a) in a DMSO solution of BzNH_2_ (**Figure** **6**).

All BZ/amine compositions polymerized at low temperature conditions a) and neat BA‐a treated at a) or b) yield very low gel contents or fully dissolve in DMSO. This is in accordance with observed respective poor mechanical properties and can be explained by low degree of polymerization under these conditions. Compared to the monofunctional benzoxazine C‐a, bisfunctional BA‐a/ED600 formulations require higher polymerization temperatures for a high monomer conversion. This is probably due to a reduced molecular mobility in the emerging polymer networks. All BZ/amine polymers polymerized at conditions b) and c) yielded high to very high gel contents in DMSO indicating high degree of polymerization and crosslinking density.

All sufficiently polymerized samples show a strong decrease in gel content treated with BzNH_2_ solution compared to pure DMSO. Complete dissolution is observed for samples with high amine ratios (BA‐a/ED600 = 1:1). BA‐a/ED600 ratios of 1:0.5, 1:0.25 and 1:0 show slightly higher gel contents with little difference between the respective ratios. Also, samples polymerized at higher temperatures show higher gel contents.

Overall, a higher solubility and thus higher rate of amine exchange reactions is observed for samples with higher amine content and lower polymerization temperatures, proving higher reactivity of methylene bridges in secondary amine structures ED600NH‐BA‐a (C^ar^CH_2_NHR) compared with methylene bridges in tertiary amine structures ED600N‐(BA‐a)_2_ and poly(BA‐a) (C^ar^CH_2_NR_2_), probably due to steric hindrance and lower chain mobility in the highly crosslinked sections.

Dissolution of the conventional, non‐dynamic poly(BA‐a)^[^ ^33^ ^]^ in the presence of BzNH_2_ is in agreement with aminolysis reactions that have been described for benzoxazines with reactive amines.^[^ ^34^ ^]^ The fact that neat polybenzoxazines nevertheless do not behave as dynamic polymer networks, can therefore be explained by the absence of the nucleophilic structural motifs within the poly(BZ) structure (i.e., reactive primary or secondary amines with active hydrogen atoms) required for the dynamic exchange reaction.

The observed trends in reactivities of nucleophiles and electrophiles in the amine exchange reaction of aminoalkylated phenols support the mechanism presented in Scheme 2 and explain the increase in activation energies for lower amine contents, observed in stress‐relaxation measurements of the polymers. Lower amine contents lead to an increased formation of unreactive nucleophiles (tertiary amines) and less reactive electrophiles (C^ar^CH_2_NR_2_). Lower reactivity of the respective functional groups leads to increase in activation energies.

## 3 Conclusion

Ring opening polymerization of the bisbenzoxazine BA‐a in presence of the polyether diamine ED600 results in thermosetting structures that can undergo dynamic covalent equilibrium reactions enabling vitrimeric stress‐relaxation behavior. Activation energies of the dynamic exchange reaction increase with decreasing amine content and decrease with polymerization progress.

Structural research using model reactions with the monobenzoxazine C‐a and various amines revealed three different aminoalkylated phenols that occur at different ratios depending on stoichiometries and polymerization conditions. High polymerization temperatures and low amine contents result in high amounts of crosslinked tertiary amine structures amine‐BZ_2_ and poly(BZ) with C^ar^CH_2_NR_2_ methylene bridges, while high amine contents and low polymerization temperatures favour linear amine‐BZ structures featuring secondary amino groups and C^ar^CH_2_NHR methylene bridges with aromatic or aliphatic substituents.

Reactive nucleophilic primary and secondary amines can induce amine exchange reaction of aminoalkylated phenols in a S_N_‐like equilibrium reaction with electrophilic methylene bridges involving proton transfer from attacking amine to amino leaving group. This covalent exchange reaction is the source of the dynamic behavior of BA‐a/ED600 vitrimers and can follow either an associative (S_N_2‐like) or dissociative (S_N_1‐like) pathway depending on reaction conditions, which can probably be catalysed by protic acid. While amines reactivity correlates to their nucleophilicity, the methylene bridges can be sorted as follows C^ar^CH_2_NHBz > C^ar^CH_2_NHPh > C^ar^CH_2_NR_2_ with decreasing reactivity. That explains the trends regarding the activation energies observed in stress‐relaxation measurements.

## Conflict of Interest

The authors declare no conflict of interest.

## Supporting information



Supporting Information

## Data Availability

The data that support the findings of this study are available from the corresponding author upon reasonable request.
