# Supplementary material for: Amine Exchange of Aminoalkylated Phenols as Dynamic Reaction in Benzoxazine/Amine‐Based Vitrimers
Source: Macromol Rapid Commun. 2024 Oct 10;45(23):2400557. doi: 10.1002/marc.202400557 (PMC11628363; doi:10.1002/marc.202400557)
Supplement: Supplementary file 1 — Supporting Information [file MARC-45-2400557-s001.docx]

Supporting Information
©Wiley-VCH 2021
69451 Weinheim, Germany

Amine Exchange of Aminoalkylated Phenols as Dynamic Reaction in Benzoxazine/Amine-Based Vitrimers

Adrian Wolf,^[a,b]^ Lea Pursche,^[a,c]^ Laura Boskamp,^[a]^ Katharina Koschek^[a,c]^*

[a] A. Wolf, Dr. L. Boskamp, Dr. K. Koschek
Fraunhofer Institute for Manufacturing Technology and Advanced Materials (IFAM)
Wiener Straße 12, 28359, Bremen, Germany
E-mail: katharina.koschek@ifam.fraunhofer.de

[b] A. Wolf
Department 2 Biology/Chemistry
University of Bremen

Leobener Straße 3, 28359 Bremen, Germany

[c] Lea Pursche, Dr. K Koschek

Faculty of Production Engineering
University of Bremen
Bibliothekstraße 1, 28359 Bremen, Germany

**Abstract:** Bisfunctional benzoxazine and polyether diamine-based polymers show Arrhenius-like stress-relaxation varying with stoichiometry and polymerization temperatures proving vitrimeric behavior. Molecular structural investigations reveal the presence of different aminoalkylated phenols occurring at varying ratios depending on polymer composition and polymerization conditions. The vitrimeric mechanism was found to involve an amine exchange reaction of aminoalkylated phenols in an equilibrium reaction like a nucleophilic substitution reaction. As determined by molecular studies and dissolution experiments in reactive solvents, aliphatic and aromatic primary as well as aliphatic secondary amines in the polybenzoxazine structure can act as nucleophiles in reaction with electrophilic methylene bridges. Thus, aminoalkylated phenols proved to be a relevant structural motif resulting a vitrimeric polybenzoxazine due to amine exchange reaction.

DOI: 10.1002/anie.2021XXXXX

Table of Contents

**1. Experimental Procedures**

**2. Supplementary Data (Chapter 2 Results and Discussion)**

**2.1 Supplementary Data (Chapter 2.1 Vitrimeric Behavior of BZ/Amine Thermosets)**

**2.2 Supplementary Data (Chapter 2.2 Polymerization and Structure Elucidation of Benzoxazine/Amine Polymers)**

**2.3 Supplementary Data (Chapter 2.3 Dynamic Bond Exchange Mechanism in Benzoxazine/Amine Polymer Networks)**

**3. References**
**4. Author Contributions**

1. Experimental Procedures

**BA-a/ED600 polymerization**

The polymers were polymerized following the established procedures.^[29]^ Different stoichiometric ratios of BA-a/polyetheramine ED600 were prepared: 1:1, 1:0.5, 1:0.25, 1:0.125, 1:0 (neat BA‑a). The mixtures were liquefied and homogenized at 120 °C within 15 min and then poured onto silicone coated paper or into silicone moulds (1 cm x 3.5 cm). For each ratio, one sample was polymerized according to the following conditions: a) 120 °C (2 h); b)120 °C (2 h), 150 °C (2 h); c)120 °C (2 h), 150 °C (2 h), 180 °C (2 h). For the 1:0 ratio an additional sample was heated according to the manufacturer information: d) 180 °C (2 h), 200 °C (2 h). All reactions yielded yellow to red solids.

**Stress-relaxation measurements**

The following polymer types were used to perform stress-relaxation measurements: BA-a/ED600(1:1)-b,c, BA-a/ED600(1:0.5)-b,c, BA‑a/ED600(1:0.25)-b,c. Stress-relaxation experiments were performed on a Discovery Hybrid Rheometer (TA Instruments, New Castle, Delaware, US) using rectangular samples sized 3.5 cm ∙ 1 cm ∙ 0.3 cm. For each polymer type, one sample was heated to the respective temperature (120 °C, 130 °C, 140 °C or 150 °C), a torsional strain of 1 % was applied and the flexural modulus G(t) was recorded over time. The relaxation times $\tau^{*}$ were defined as the time where G(t) was reduced to 1/e of the initial modulus G(1 s). $\ln(\tau^{*}\left( T \right))$ was plotted against inverse 1000 ∙ temperature $T$ according to linearized Arrhenius equation (Equation 1). The slope of the linear fit was used to determine the activation energies $E_{a}$ of the exchange reaction using Equation 2 with $R$ being the universal gas constant.

$\ln(\tau^{*}\left( T \right))=ln\tau^{0}+\frac{E_{a}}{R}\cdot\frac{1}{T}$ (Equation 1)

$E_{a}=slope from linear fit\cdot R$ (Equation 2)

**NMR measurements**

All NMR-spectra were recorded in DMSO-d_6_, using a 600 MHz spectrometer (AVANCE NEO 600 MHz, Bruker Corporation, Massachusetts, US). The residual solvent signals were used to reference the spectra.

**IR measurements**

All IR-spectra were recorded using an ATR spectrometer (ALPHA II, Bruker Corporation, Massachusetts, US). Recording range 400‑4000 cm^-1^; resolution ± 2 cm^-1^, 32 scans.

**DSC measurements**

Thermal behaviour of the reactions was analyzed using a differential scanning calorimeter (Discovery DSC, TA Instruments, New Castle, Delaware, US). The samples weighed 1.5–3.0 mg and were heated from -20–350 °C at a rate of 10 K/min.

**TGA-IR measurements**

TGA-IR data were recorded using a simultaneous thermogravimetric analysis (STA) machine (NETZSCH STA 449F3, Netzsch, Selb, Germany). The samples weighed 6–8 mg and were heated from 35–550 °C at a rate of 10 K/min under an atmosphere of argon.

**Chemicals**

Aniline, polyetheramine “Jeffamine” M600, polyetheramine “Jeffamine” ED600, benzylamine, dibenzylamine, diphenylamine, *N*,*N*-dimethylbenzylamine, silica gel, *n*‑hexane and *para*-cresol were purchased from Merck (Darmstadt, Ger.). DMSO d_6_ and CDCl_3_ were provided by Deutero (Kastellaun, Ger.). Na_2_SO_4_, ethanol, paraformaldehyde were obtained from Carl Roth (Karlsruhe, Ger.). BA-a was obtained from Huntsman (Texas, US). Chloroform and NaOH were purchased from VWR (Pennsylvania, US).

**Synthesis of benzoxazine based on *para*-cresol and aniline (C-a)**

C-a was synthesized following our previously established procedure.^[29,35]^ Aniline (23.5 g, 252 mmol), paraformaldehyde (16.7 g, 556 mmol) and *para*-cresol (30.0 g, 277 mmol) were reacted at 100 °C for 1 h under nitrogen atmosphere. Workup was performed by dissolving in 350 ml chloroform and washing three times with aqueous sodium hydroxide solution (3∙150 ml) (1mol/l). Further purification was achieved by recrystallization from ethanol. The product was obtained as white solid, 47.8 g (yield = 67 %).


^1^H-NMR (600 MHz, DMSO-d_6_, 296 K): δ / ppm = 7.23 (t, *J* = 8.0 Hz, 2H, H^h^), 7.11 (d, *J* = 7.7 Hz, 2H, H^g^), 6.91 (s, 1H, H^d^), 6.88 (d, *J* = 8.2 Hz, 1H, H^b^), 6.85 (t, *J* = 7.3 Hz, 1H, H^i^), 6.62 (d, *J* = 8.2 Hz, 1H, H^c^), 5.40 (s, 2H, H^e^), 4.60 (s, 2H, H^f^), 2.19 (s, 1H, H^a^). ^13^C{^1^H}-NMR (151 MHz, DMSO-d_6_, 296 K): δ / ppm = 151.7 (C^j^), 147.9 (C^k^), 129.1, 128.1, 127.3, 120.9, 120.4, 117.3, 115.9 (C^ar^), 78.6 (C^e^), 48.9(C^f^), 20.2 (C^a^). IR-ATR: wavenumber / cm-1: 3042 (C^ar^H), 3027 (C^ar^H), 3013 (C^ar^H), 2914, (CH_2_/CH_3_), 1600, 1579, 1493 (PhR_3_), 1454, 1446, 1356, 1220 (C^ar^OCH_2_), 1204, 1168, 1141, 1119, 1042, 942 (oxazine ring), 915.

**Determination of polymerization kinetics of C-a with amines**

C-a (0.500 g, 2.22 mmol) and 1 equivalent of the respective amine (polyetheramine M600 (M600NH_2_), benzylamine (BzNH_2_), aniline (PhNH_2_), diphenylamine (Ph_2_NH), dibenzylamine (Bz_2_NH), *N*,*N*-dimethylbenzylamine (Me_2_NBz)) were reacted 120 °C under constant stirring. In regular intervals from 2 min to 6 h, samples were taken and dissolved in DMSO-d_6_. The conversion of C-a was determined from the respective ^1^H-NMR spectra.


^1^H-NMR (600 MHz, DMSO-*d*_6_) δ / ppm = 10.70 (s, 1H, H^r^), 7.02 (t, *J* = 7.8 Hz, 2H, H^m^), 6.91 (s, 1H, H^l^), 6.70 (s, 1H, H^k^), 6.56 (d, *J* = 8.1 Hz, 2H, H^g^), 6.48 (t, *J* = 7.1 Hz, 1H, H^h^), 5.94–5.85 (m, 1H, H^p^), 5.00–4,89 (m, 1H, H^q^), 4.14 (d, *J* = 5.9 Hz, 2H, H^c^), 3.95–3.85 (m, 2H, H^d^), 3.60–3.27 (m, CH_1/2_O), 3.25 (s, 3H, H^f^), 2.84–2.72 (m, 1H, H^e^), 2.12 (s, 3H, H^b^), 1.14–0.97 (m, CHC*H*_3_). ^13^C{^1^H}-NMR (151 MHz, DMSO-d_6_, 298 K): δ / ppm = 153.5 (1C, C^o^), 149.0 (1C, C^n^), 128.8 (2C, C^m^), 127.0 (1C, C^l^), 127.1 (1C, C^k^), 126.1 (1C, C^j^), 125.5 (1C, C^i^), 115.5 (1C, C^h^), 111.9 (2C, C^g^), 75.8, 74.5, 74.3, 74.2, 73.8, 72.2, 71.3, 70.4, 67.9 (CH_1/2_O), 58.4 (1C, C^f^), 51.9 (1C, C^e^), 49.2 (1C, C^d^), 41.0 (1C, C^c^), 20.3 (1C, C^b^), 17.2 (1C, C^a^).


^1^H-NMR (600 MHz, DMSO-d_6_, 298 K): *δ* / ppm = 10.63 (s, 1H, H^u^), 7.39–7.18 (m, 6H, H^j,m,n^), 7.02 (t, *J* = 6.9 Hz, 2H, H^o^), 6.93 (s, 1H, H^k^), 6.70 (s, 1H, H^l^), 6.57 (d, *J* = 7.6 Hz, 2H, H^e^), 6.49 (t, *J* = 7.3 Hz, 1H, H^f^), 5.94 (t, *J* = 6.0 Hz, 1H, H^s^), 4.15 (d, *J* = 5.9 Hz, 2H. H^b^), 3.85 (s, 2H, H^c^), 3.70 (s, 2H, H^d^), 3.62 (s, 1H, H^t^), 2.13 (s, 3H, H^a^). ^13^C{^1^H}-NMR (151 MHz, DMSO-d_6_, 298 K): *δ* / ppm = 153.5 (1C, C^r^), 148.9 (1C, C^q^), 148.9 (1C, C^p^), 148.9 (2C, C^o^), 128.4 (2C, C^n^), 128.3 (2C, C^m^), 127.4 (1C, C^l^), 127.3 (1C, C^k^), 127.0 (1C, C^j^), 126.3 (1C, C^i^), 125.6 (1C, C^h^), 122.2 (1C, C^g^), 115.5 (1C, C^f^), 112.1 (2C, C^e^), 51.5 (1C, C^d^), 50.9 (1C, C^c^), 41.0 (1C, C^b^), 20.4 (1C, C^a^).


^1^H-NMR (600 MHz, DMSO-d_6_, 298 K): δ / ppm = 9.21 (s, 1H, H^h^), 7.04–7.01 (m, 4H, H^g^), 6.92 (s, 2H, H^f^), 6.59 (d, *J* = 7.5 Hz, 4H, H^e^), 6.53 (t, *J* = 7.3 Hz, 2H, H^d^), 5.98 (t, *J* = 5.9 Hz, 2H, H^c^), 4.21 (d, *J* = 5.8 Hz, 4H, H^b^), 2.12 (s, 3H, H^a^).


^1^H-NMR (600 MHz, DMSO-*d*_6_) δ / ppm = 10.63 (s, 1H, H^k^), 7.38–7.29 (m, 10H, C^ar^H), 7.02 (t, 2H, H^j^), 6.93 (s, 1H, H^i^), 6.70 (s, 1H, H^h^), 6.57 (d, *J* = 7.6 Hz, 2H, H^g^), 6.48 (t, 1H, H^f^), 5.92 (t, *J* = 5.9 Hz, 1H, H^e^), 4.15 (d, *J* = 5.9 Hz, 2H, H^d^), 3.84 (s, 2H, H^c^), 3.70 (s, 4H, H^b^), 2.12 (s, 3H, H^a^).

**Synthesis of BzNH-C-a**

C-a (5.00 g, 22.2 mmol) and benzylamine (2.39 g, 22.2 mmol) were stirred in an oil bath at 120 °C for 2 h. The resulting yellow oil was further purified via silica gel column chromatography (silica gel, pore size 60 Å, 230–400 mesh particle size, 40–63 µm particle size; gradient elution ethyl acetate (5–50 %) in *n*-hexane. The desired product eluded in the last fraction. R_f_ = 0.11 (ethyl acetate in *n*-hexane 15 %). After drying under reduced pressure, the product was obtained as brown, highly viscous oil.

**Investigation of dynamic reaction**

1 equivalent of polyetheramine M600 (0.280 g, 4.66 mmol) was added to BzNH-C-a (0.155 g, 4.66 mmol) and the mixture was reacted at 120 °C under constant stirring. In regular intervals from 2 min to 24 h, samples were taken and dissolved in DMSO-d_6_. The conversion of BzNH-C-a and the yield of M600NH-C-a was determined from the respective ^1^H-NMR spectra.

**Determination of yield and conversion from ^1^H-NMR spectra**

The quantities of all C-a derivatives were determined from the respective integrals of the signals in ^1^H-NMR spectra. The sum of all integrals corresponding to C(ar)*C*H_3_ groups (1.90–2.25 ppm) were used as internal standard. The quantity of the respective compound was determined using the integral of the best separated signal. The following signals were used here. C-a: 5.40 ppm (s, 2H); BzNH‑C‑a: 3.84 ppm (s, 2H); M600NH-C-a (ring-opening reaction): 4.13 ppm (d, 2H), M600NH-C-a (dynamic reaction): 2.76 ppm (quin, 1H); M600N-(C‑a)_2_ 4.10 ppm (s, 4H); PhNH-C-a: 4.22 ppm (d, 4H); BzNH-C-a. Equation 1 was used to calculate the respective yield or conversion. $N$ = number of H-atoms in the molecule of interest corresponding to the respective signal in ^1^H-NMR spectrum.

$yield=\left( 100 \%-conversion \right)=\frac{\frac{1}{N_{H, compound signal}}\cdot\int compound signal}{\frac{1}{N_{H, C(ar)CH_{3}}}\cdot\sum\int signals of all C(ar)CH_{3}}\cdot100 \%$ (1)

**Solubility experiments**

The BA-a/ED600 polymers were manually crushed into small shards with diameters of 1-5 mm. From these fragments, 296–305 mg were placed in a test tube and immersed in 3 mL of either pure DMSO, or a solution of the respective reactive compound in DMSO (1 mol/l). The reactive compounds were either benzylamine, aniline, diphenylamine, dibenzylamine, *N*,*N*-dimethylbenzylamine, or *para*-cresol. The open test tubes were then placed in a pre-heated convection oven at 120 °C for 2 h. The contents were then poured into a beaker with chloroform (15 ml), stirred and filtered. The obtained residues were washed with chloroform (3∙10 ml) and dried to mass constancy in an oven at 140 °C to mass constancy.

2. Supplementary Data (Chapter 2 Results and Discussion)

**2.1 Supplementary Data (Chapter 2.1 Vitrimeric Behavior of Benzoxazine/Amine Networks)**


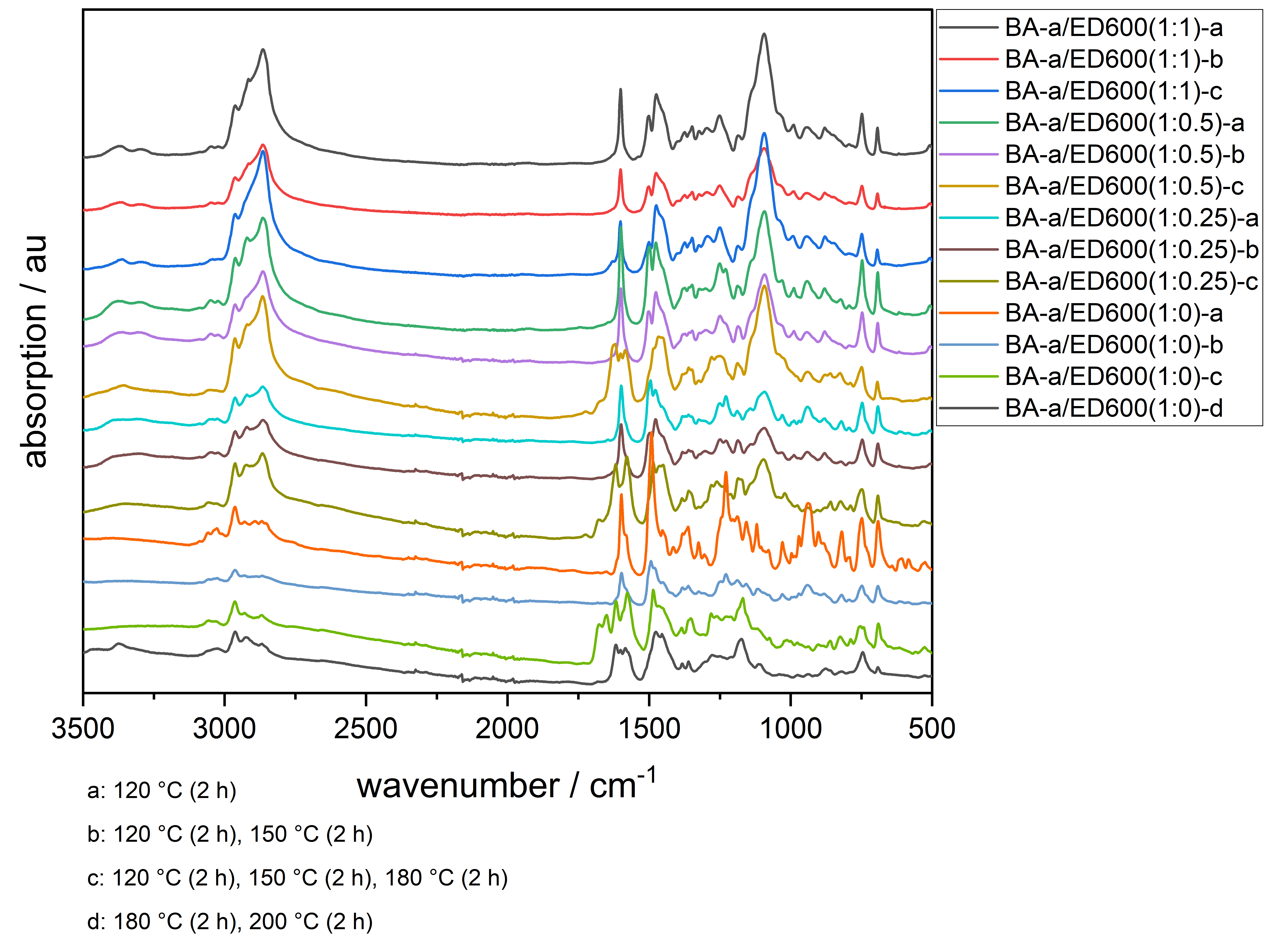


**Figure S1**: IR-ATR Spectra: Mixtures of bisbenzoxazine BA-a and polyether diamine ED600 in different ratios BA-a/ED600 (1:x) polymerized at different conditions.

**
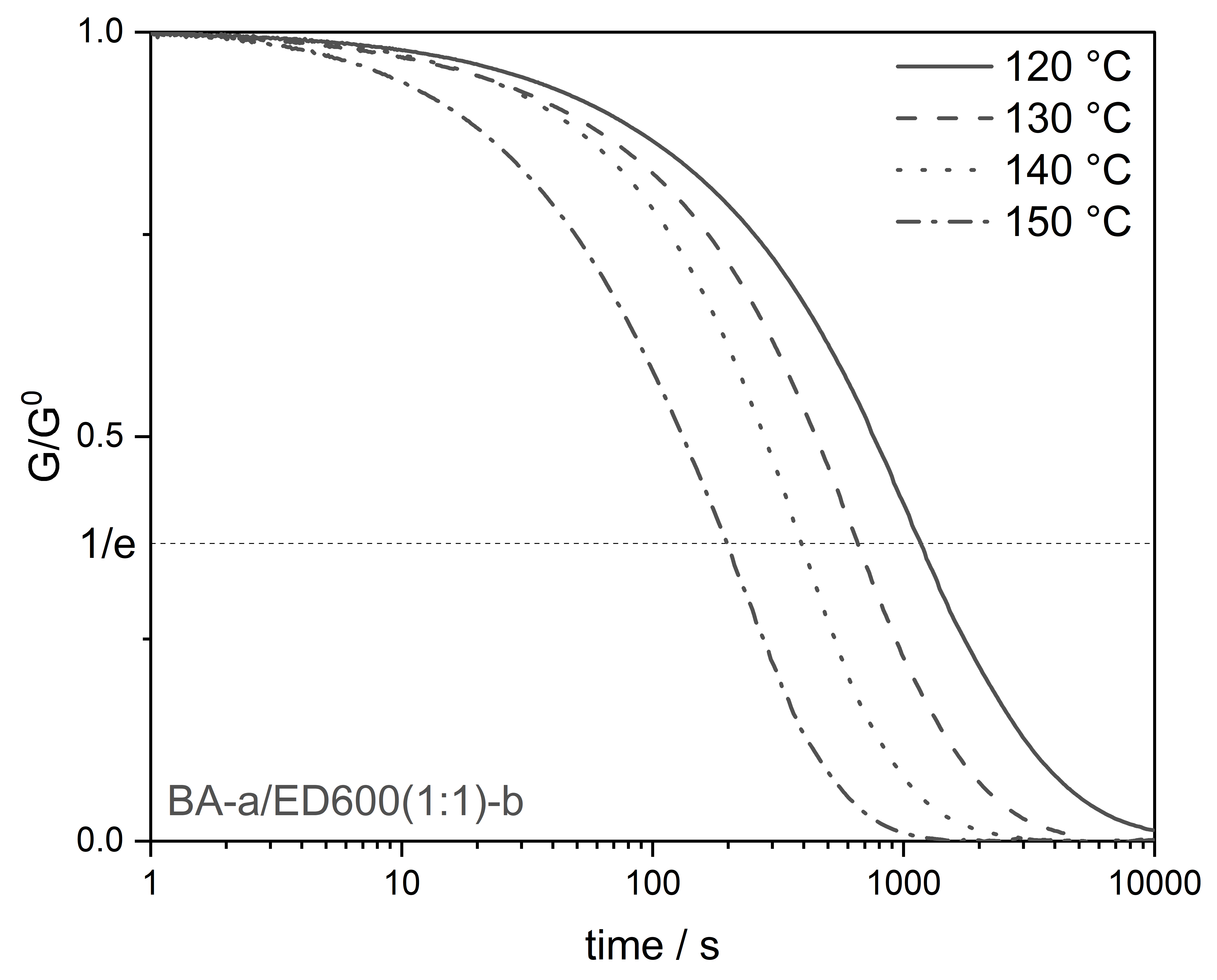
**

**Figure S2**: Stress-relaxation data: flexural modulus of BA-a/ED600(1:1)-b at different temperatures.

**
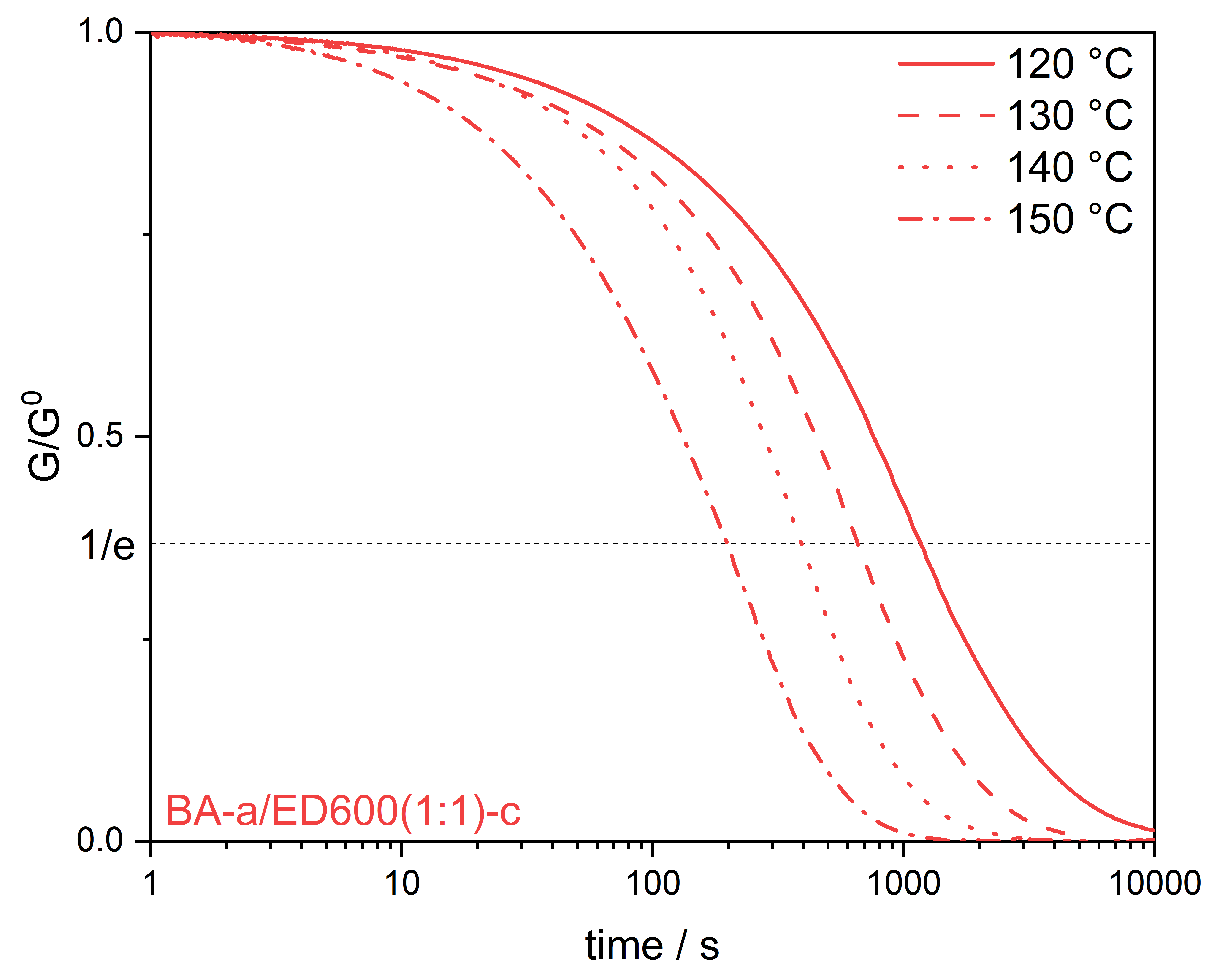
**

**Figure S3**: Stress-relaxation data: flexural modulus of BA-a/ED600(1:1)-c at different temperatures.

**
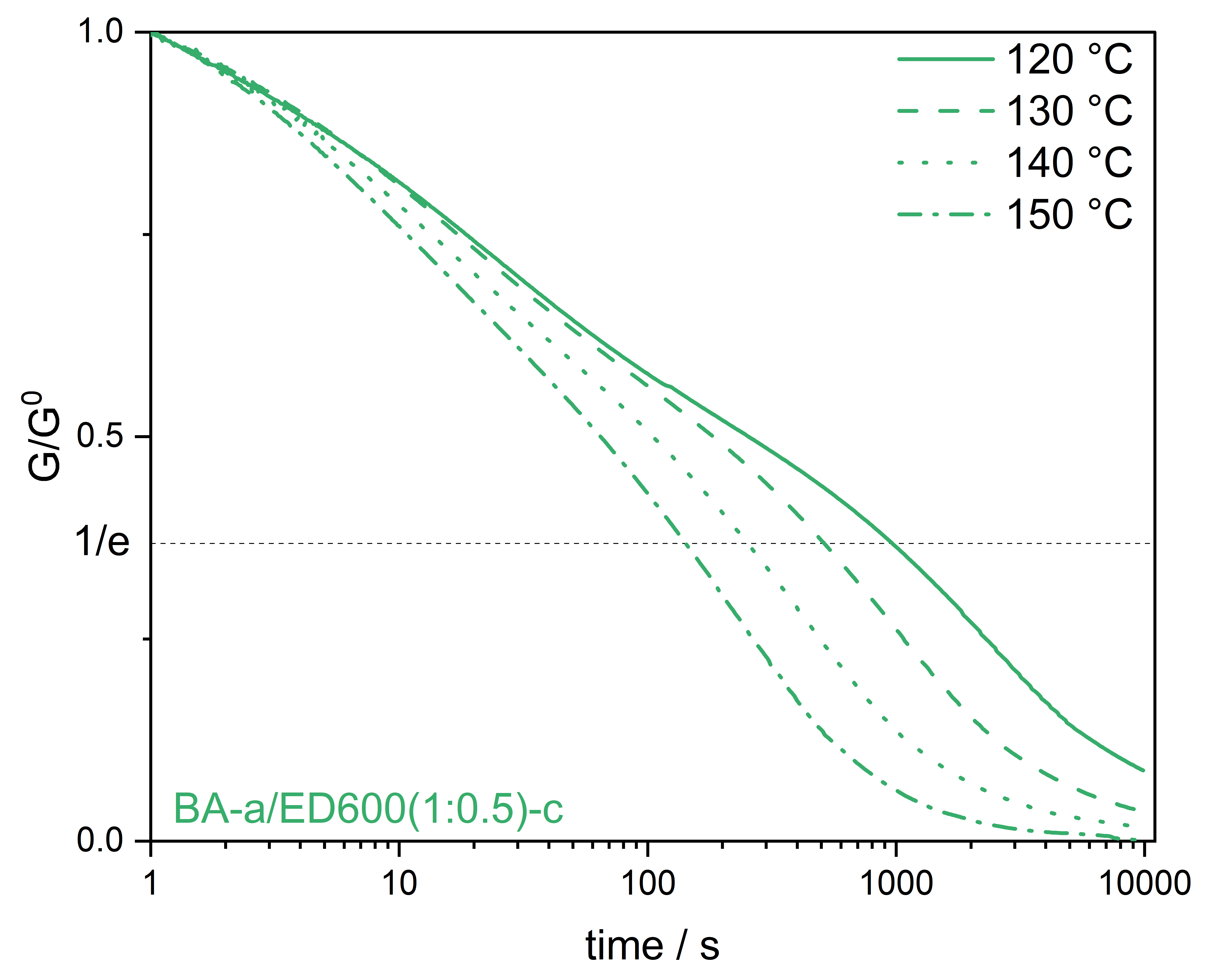
**

**Figure S4**: Stress-relaxation data: flexural modulus of BA-a/ED600(1:0.5)-c at different temperatures.

**
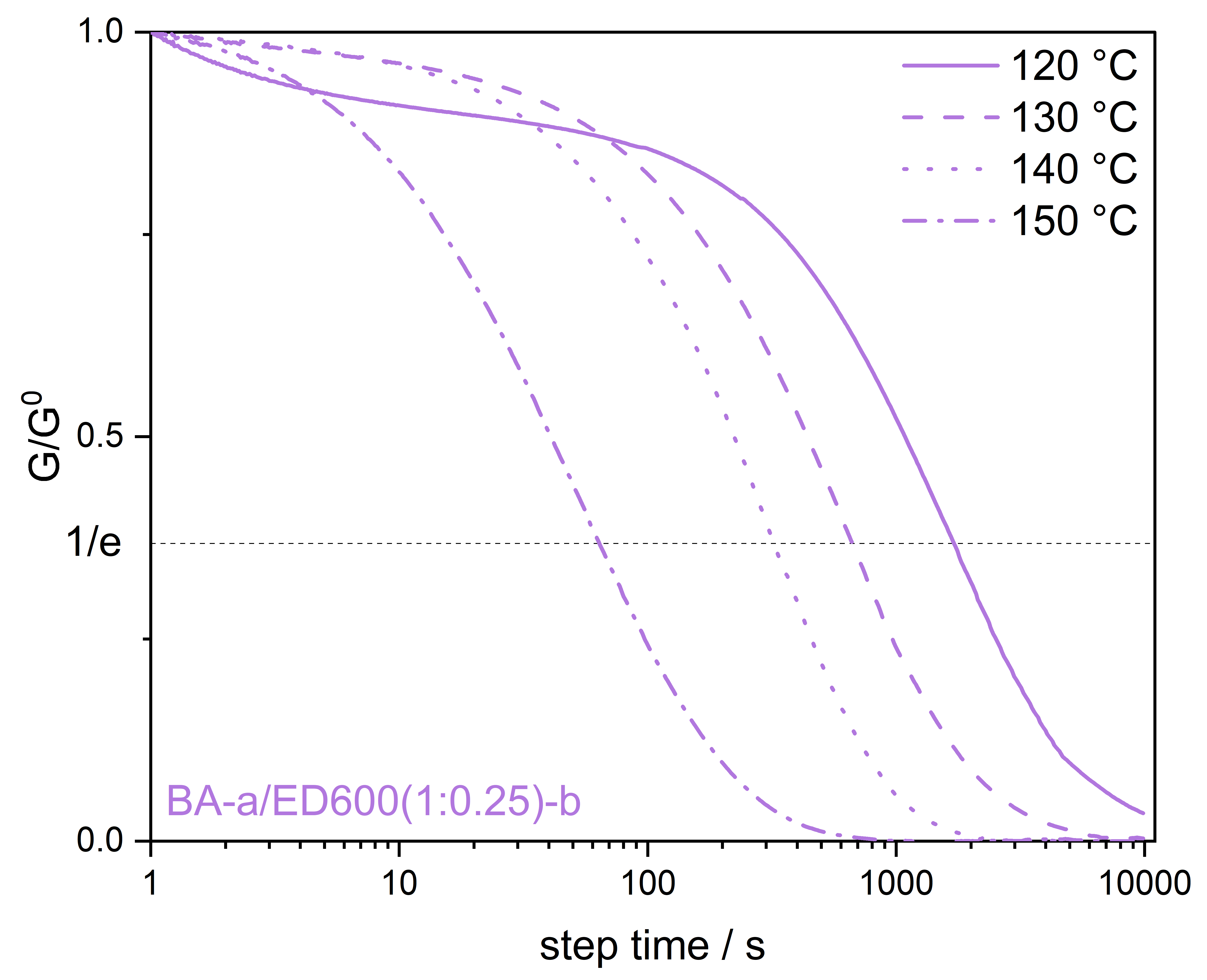
**

**Figure S5**: Stress-relaxation data: flexural modulus of BA-a/ED600(1:0.25)-b at different temperatures.

**
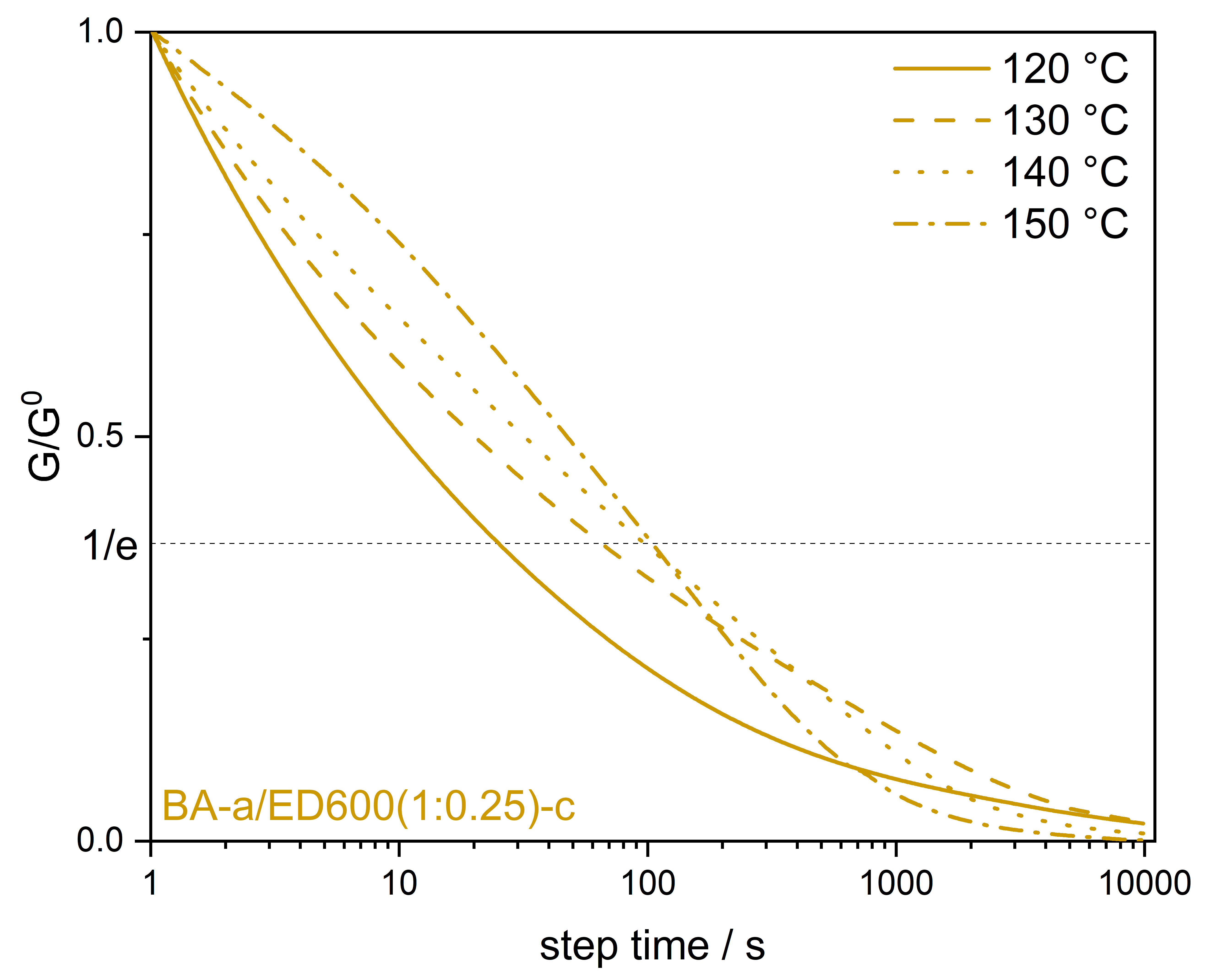
**

**Figure S6**: Stress-relaxation data: flexural modulus of BA-a/ED600(1:0.25)-c at different temperatures.


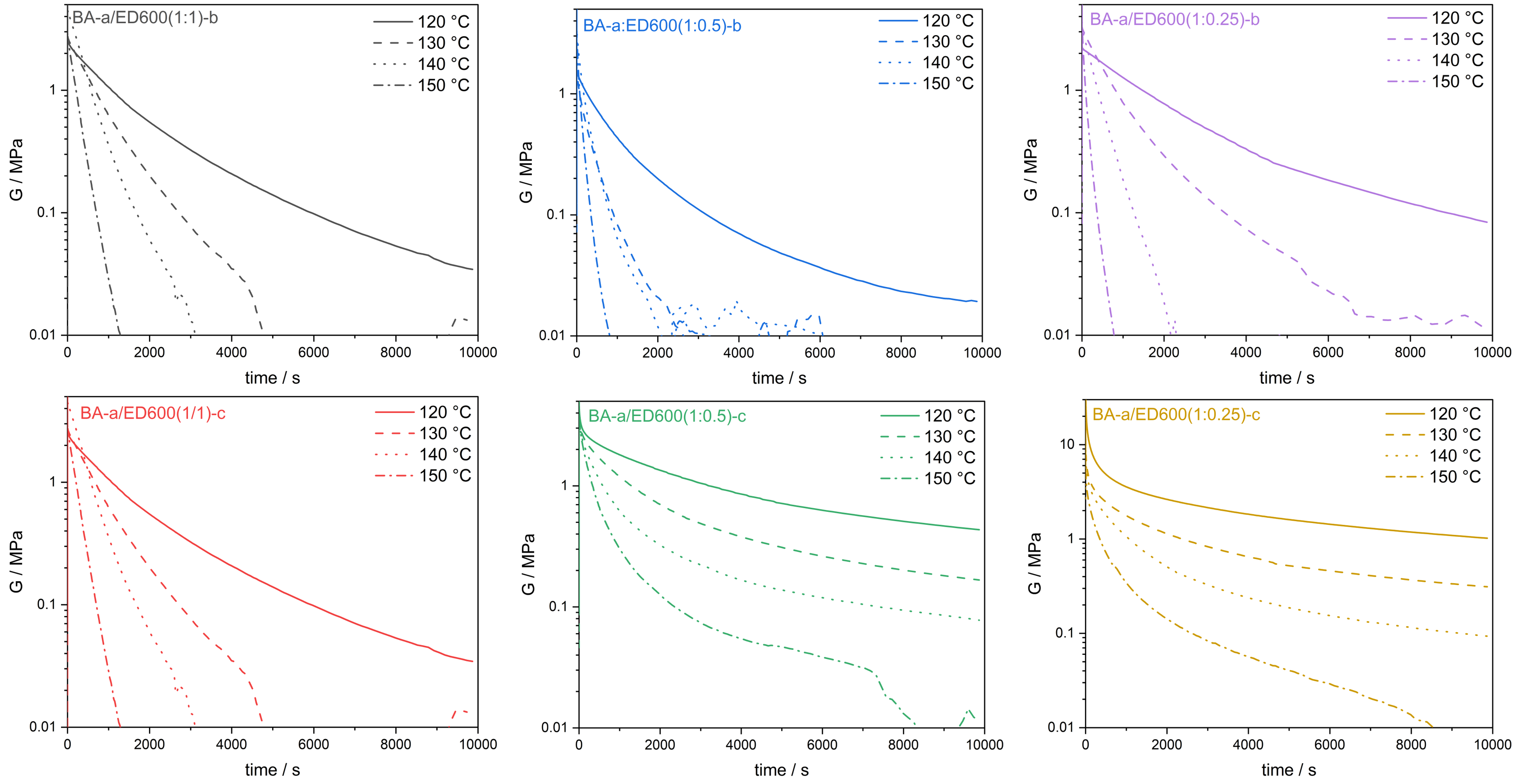


**Figure S7**: Non-normalized stress-relaxation data: flexural modulus G of BA-a/ED600 different stoichiometries polymerized at different temperatures.

**2.2 Supplementary Data (Chapter 2.2 Polymerization and Structure Elucidation of Benzoxazine/Amine Polymers)**

**Table S1**: Yields (^1^H-NMR) of C-a/M600NH_2_ bulk reactions after 120 min at 120 °C for different C-a/M600NH_2_ ratios (1:1, 1:0.5, 1:0.25) ratios and calculated M600N‑(C‑a)_2_/M600NH-C-a value. Yields were determined from 1H-NMR spectra (Figure S7).

| **molar ratio**  **C‑a/M600NH_2_** | **yield / %**  **C-a** | **yield / %**  **M600NH-C‑a** | **yield / %**  **M600N‑(C‑a)_2_** | **M600N-(C‑a)_2_/**  **M600NH-C-a** |
| --- | --- | --- | --- | --- |
| 1:1 | 16 | 59 | 13 | 0.22 |
| 1:0.5 | 24 | 36 | 14 | 0.39 |
| 1:0.25 | 47 | 18 | 11 | 0.61 |


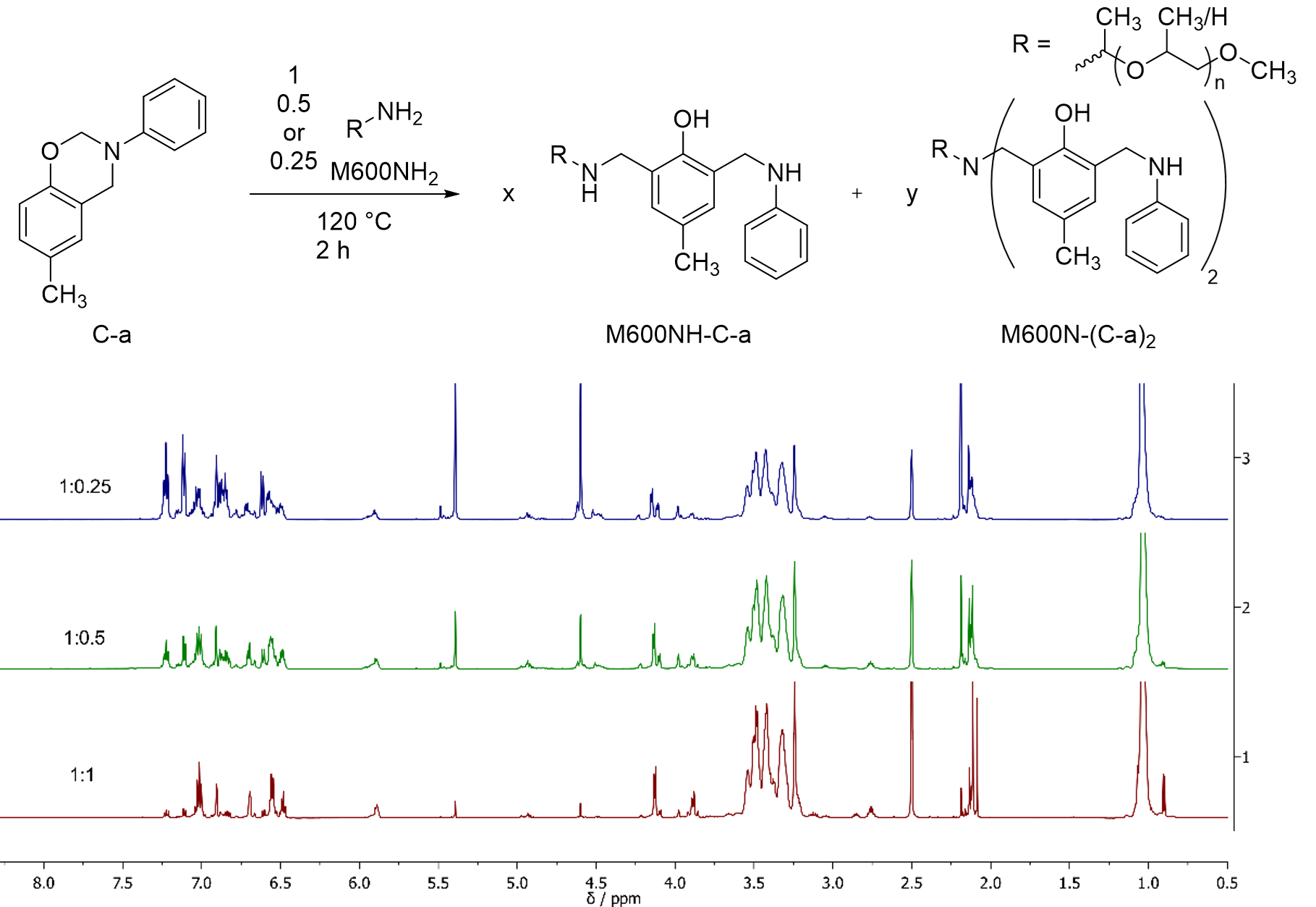


**Figure S8**: ^1^H-NMR (600 MHz, DMSO-d_6_, 296 K): Bulk reaction product (120 °C, 2 h) of C‑a with polyetheramine M600NH_2_ of different stoichiometric ratios (1:x).


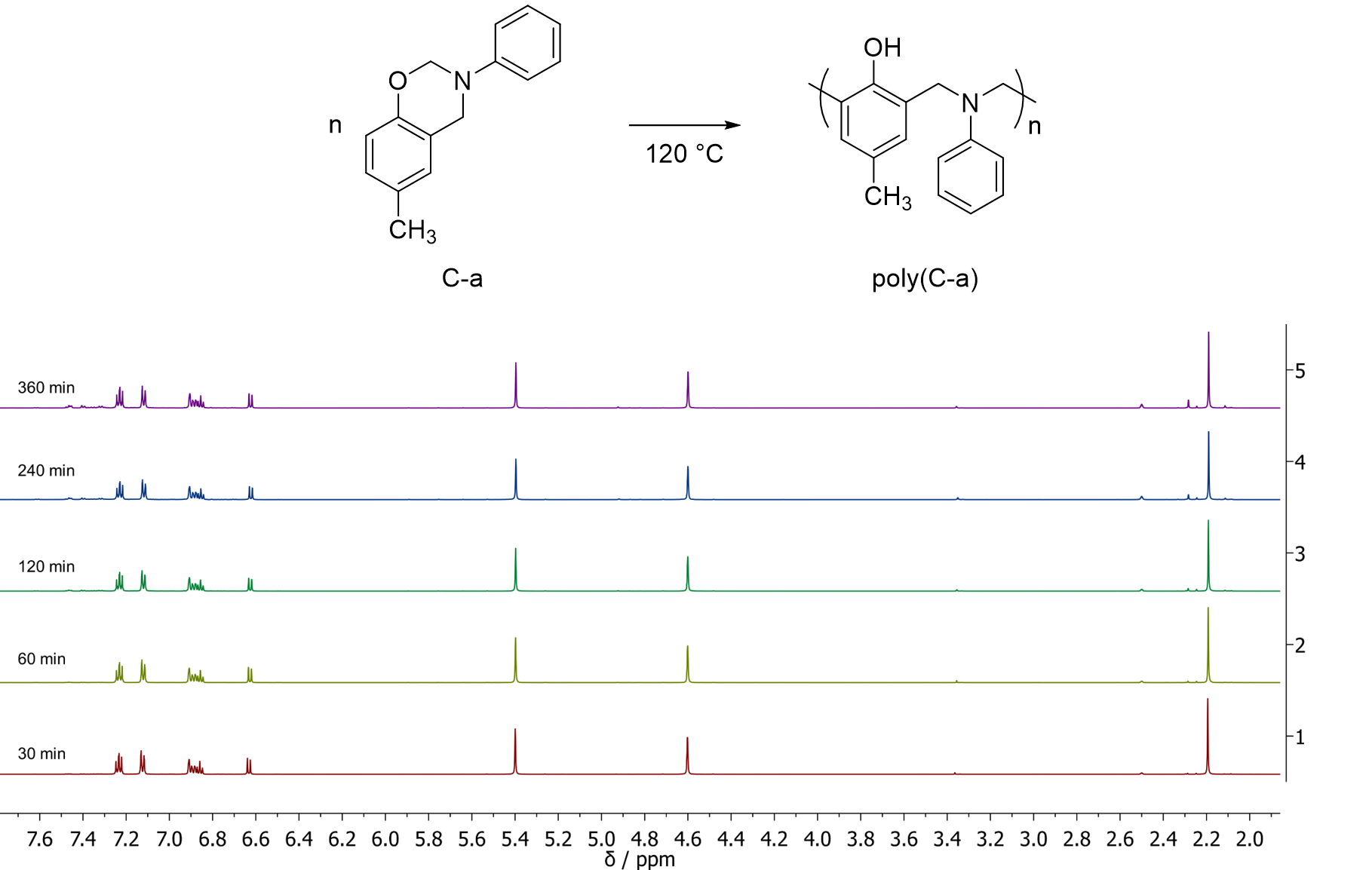


**Figure S9**: ^1^H-NMR (600 MHz, DMSO-d_6_, 296 K): Bulk reaction (120 °C) of C‑a.


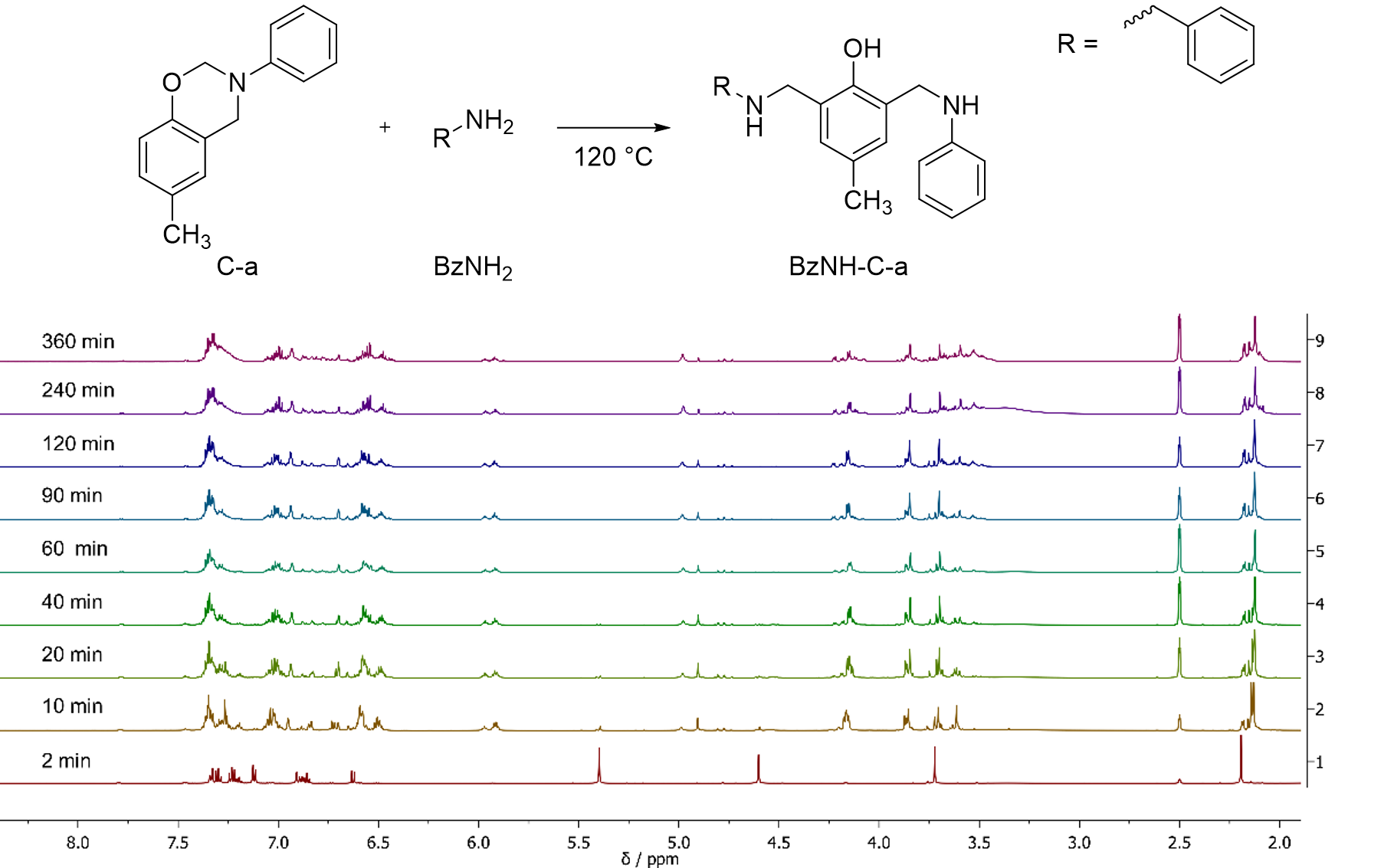


**Figure S10**: ^1^H-NMR (600 MHz, DMSO-d_6_, 296 K): Bulk reaction (120 °C) of C‑a with benzylamine BzNH_2_.


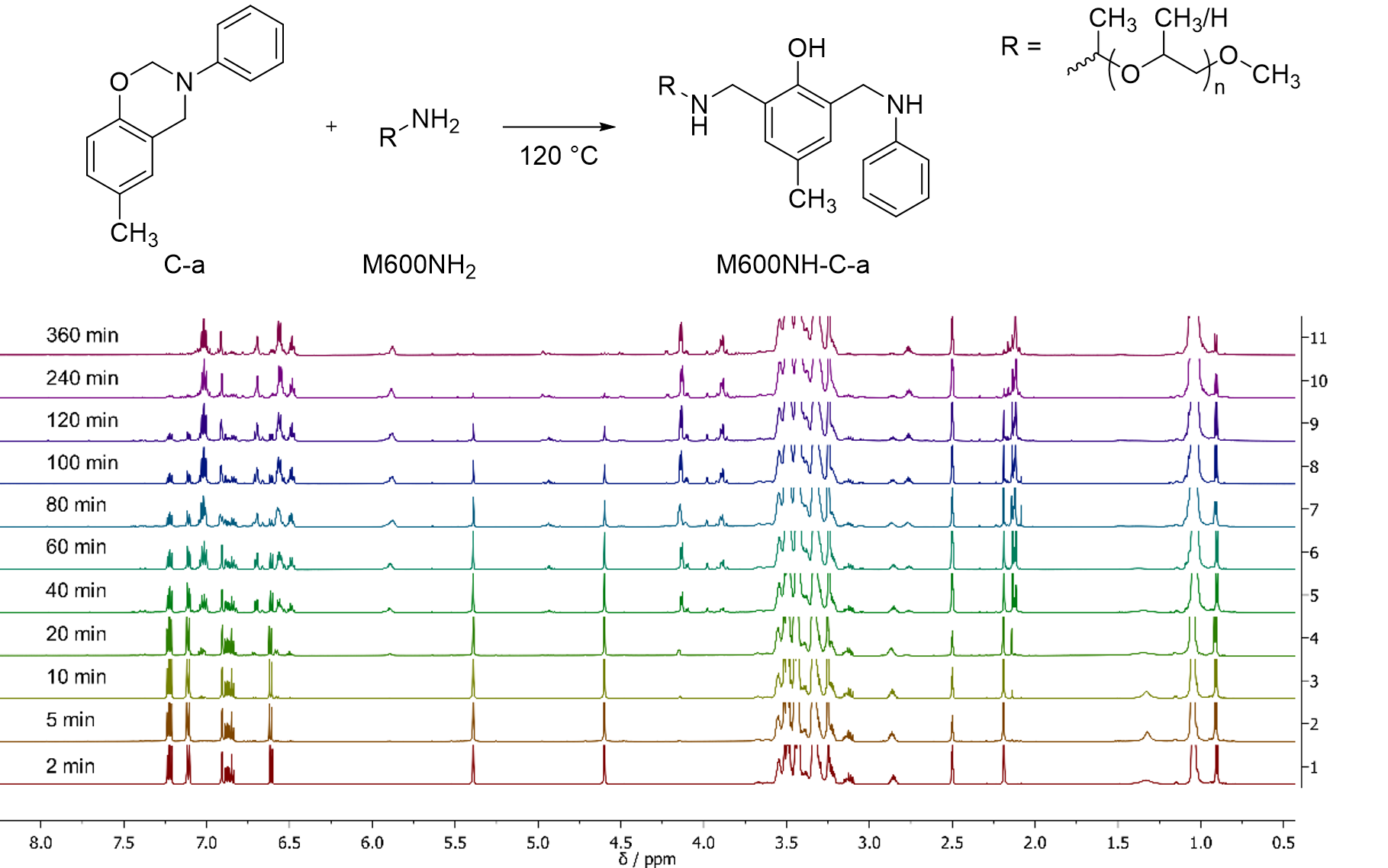


**Figure S11**: ^1^H-NMR (600 MHz, DMSO-d_6_, 296 K): Bulk reaction (120 °C) of C‑a with polyetheramine M600NH_2_.


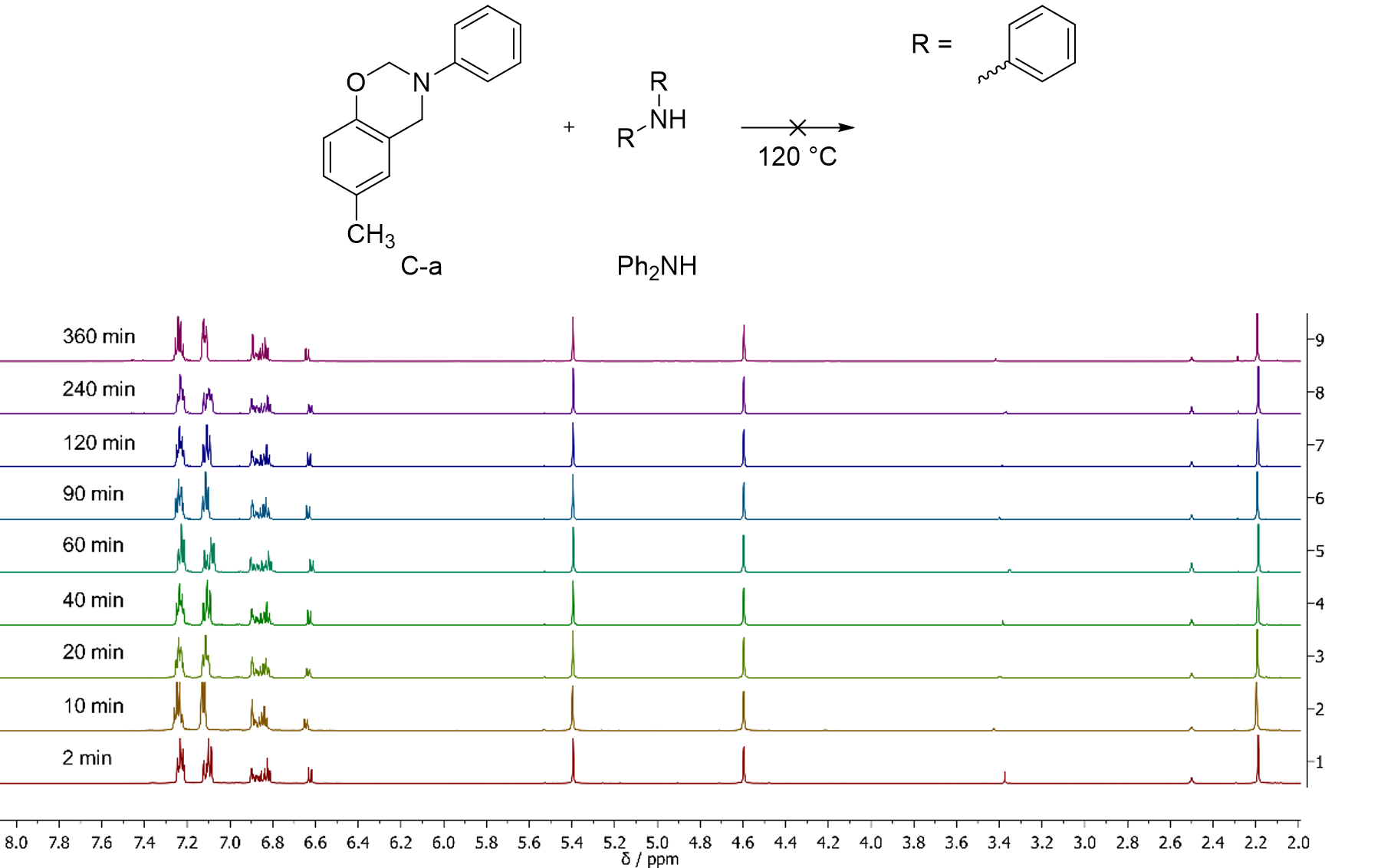


**Figure S12**: ^1^H-NMR (600 MHz, DMSO-d_6_, 296 K): Bulk reaction (120 °C) of C‑a with diphenylamine Ph_2_NH.


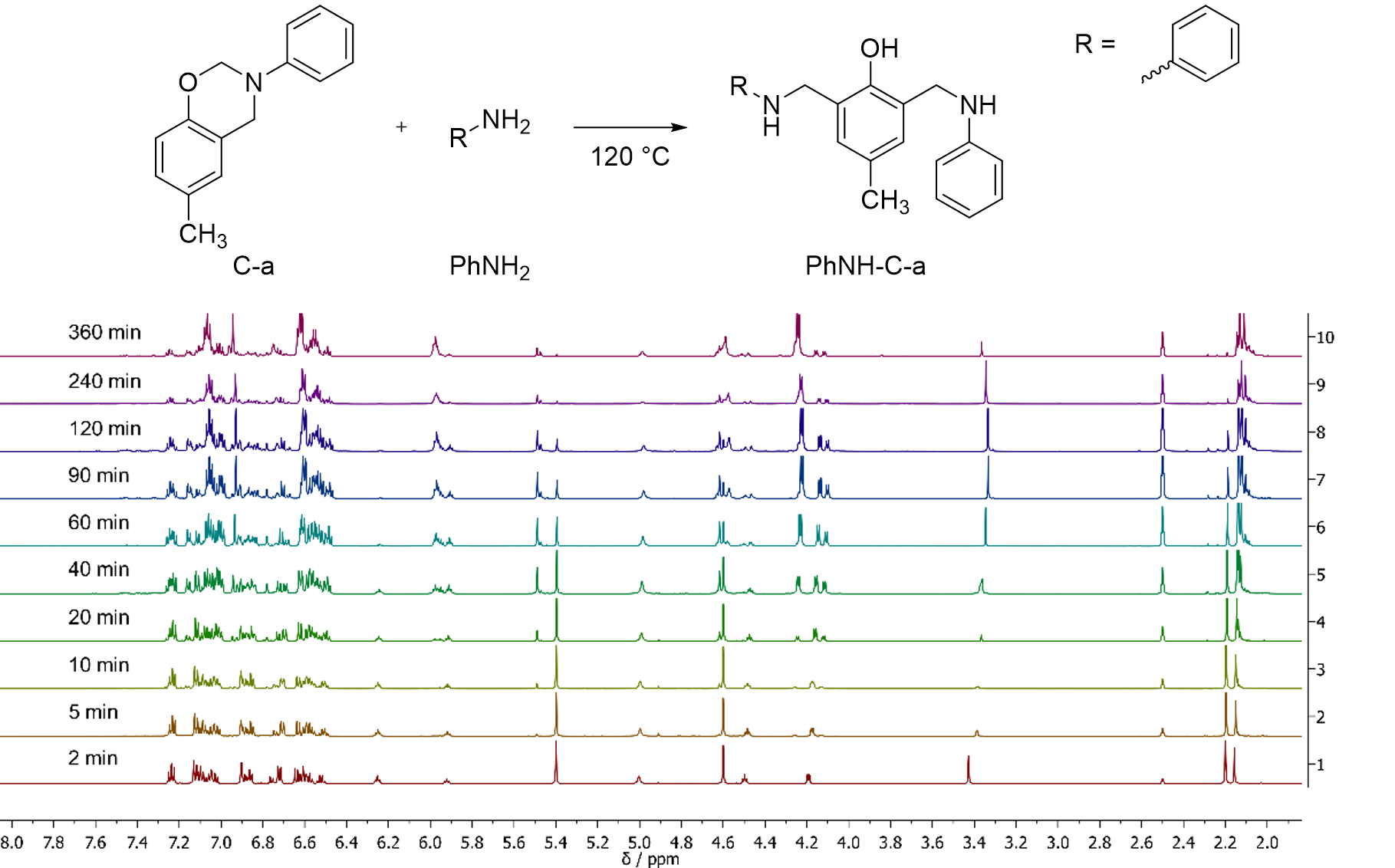


**Figure S13**: ^1^H-NMR (600 MHz, DMSO-d_6_, 296 K): Bulk reaction (120 °C) of C‑a with aniline PhNH_2_.


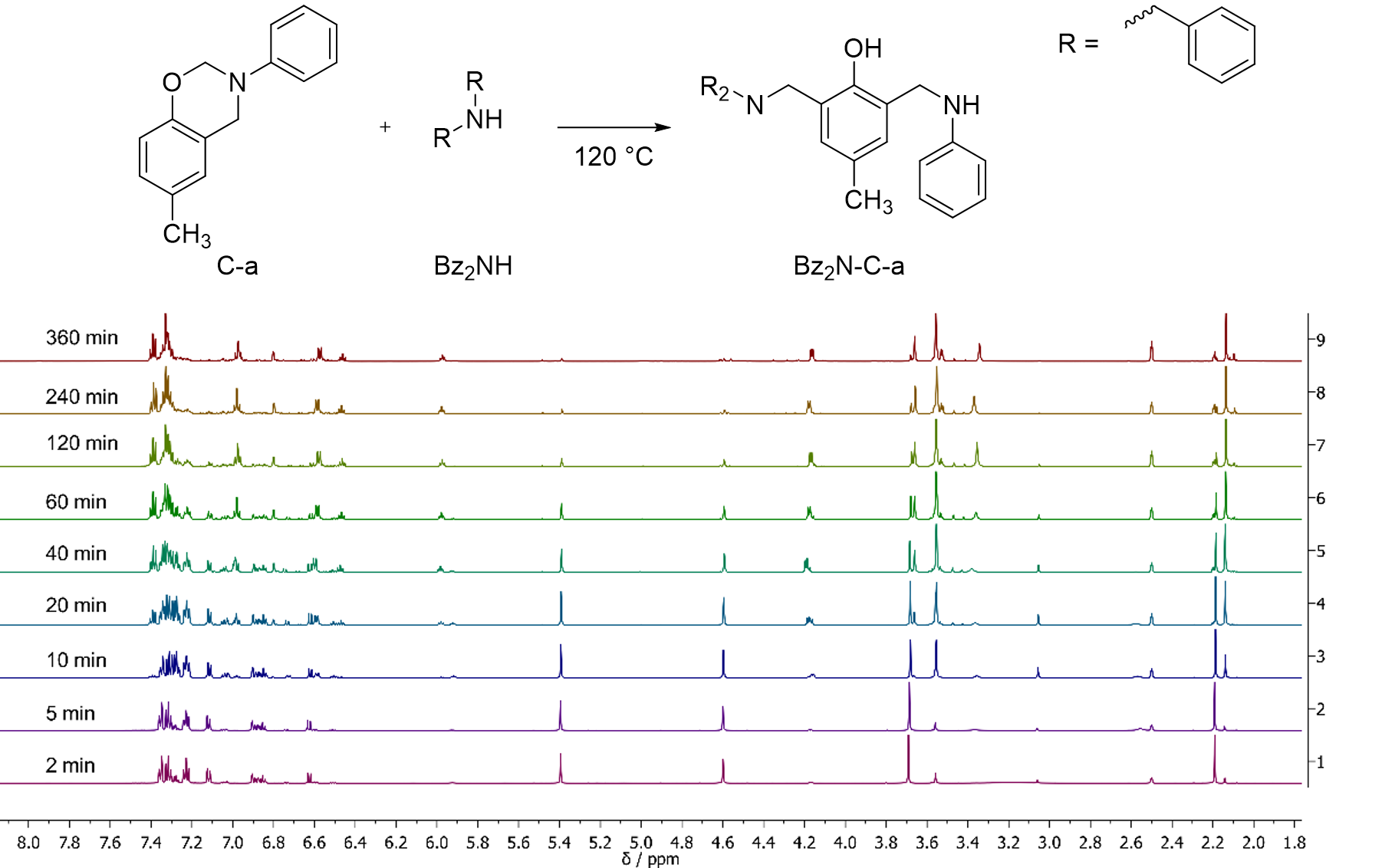


**Figure S14**: ^1^H-NMR (600 MHz, DMSO-d_6_, 296 K): Bulk reaction (120 °C) of C‑a with dibenzylamine Bz_2_NH.

**
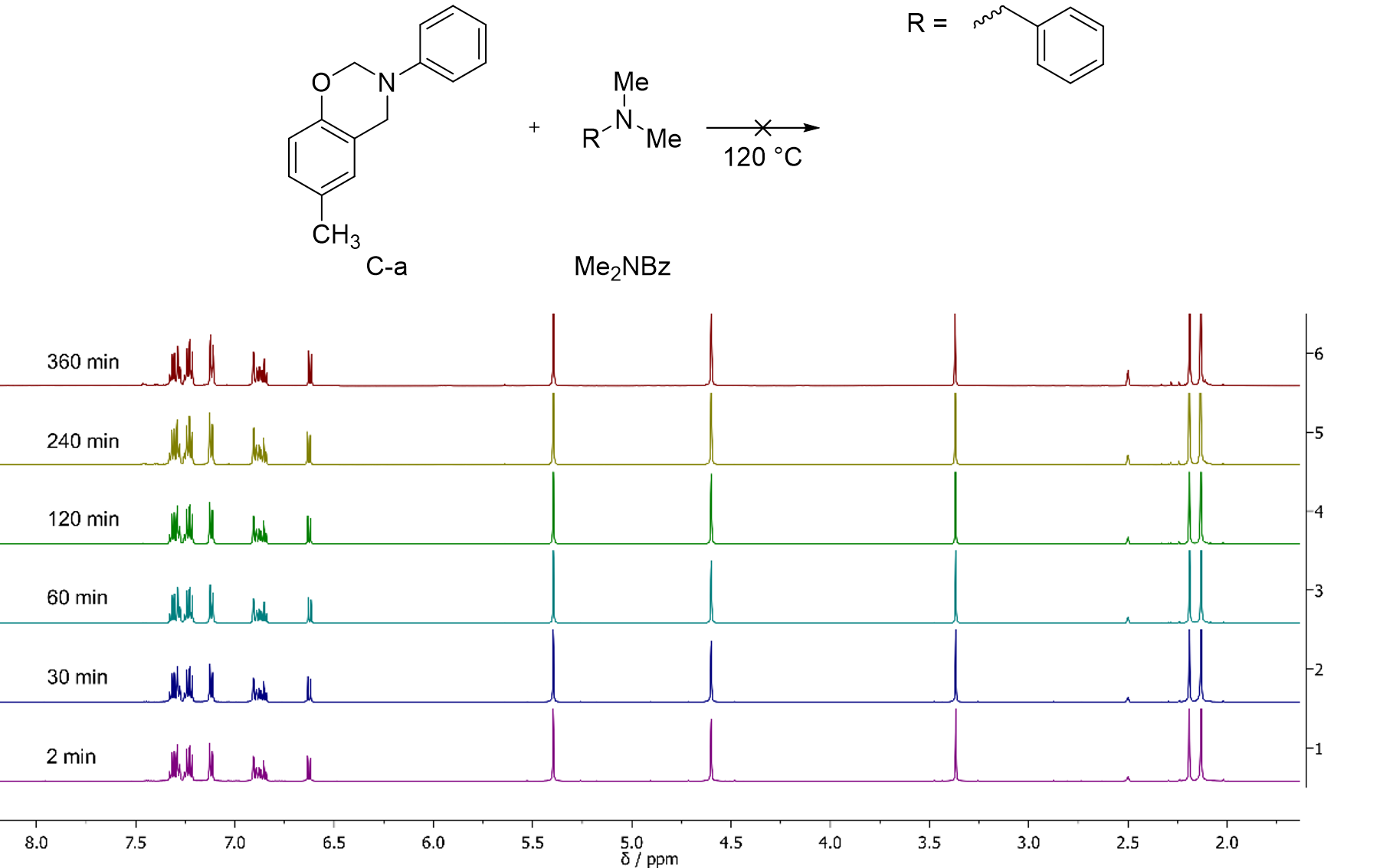
**

**Figure S15**: ^1^H-NMR (600 MHz, DMSO-d_6_, 296 K): Bulk reaction (120 °C) of C‑a with *N*,*N*-dimethylbenzylamine Me_2_NBz.

**2.3 Supplementary Data (Chapter 2.3 Dynamic Bond Exchange Mechanism in Benzoxazine/Amine Polymer Networks)**

**
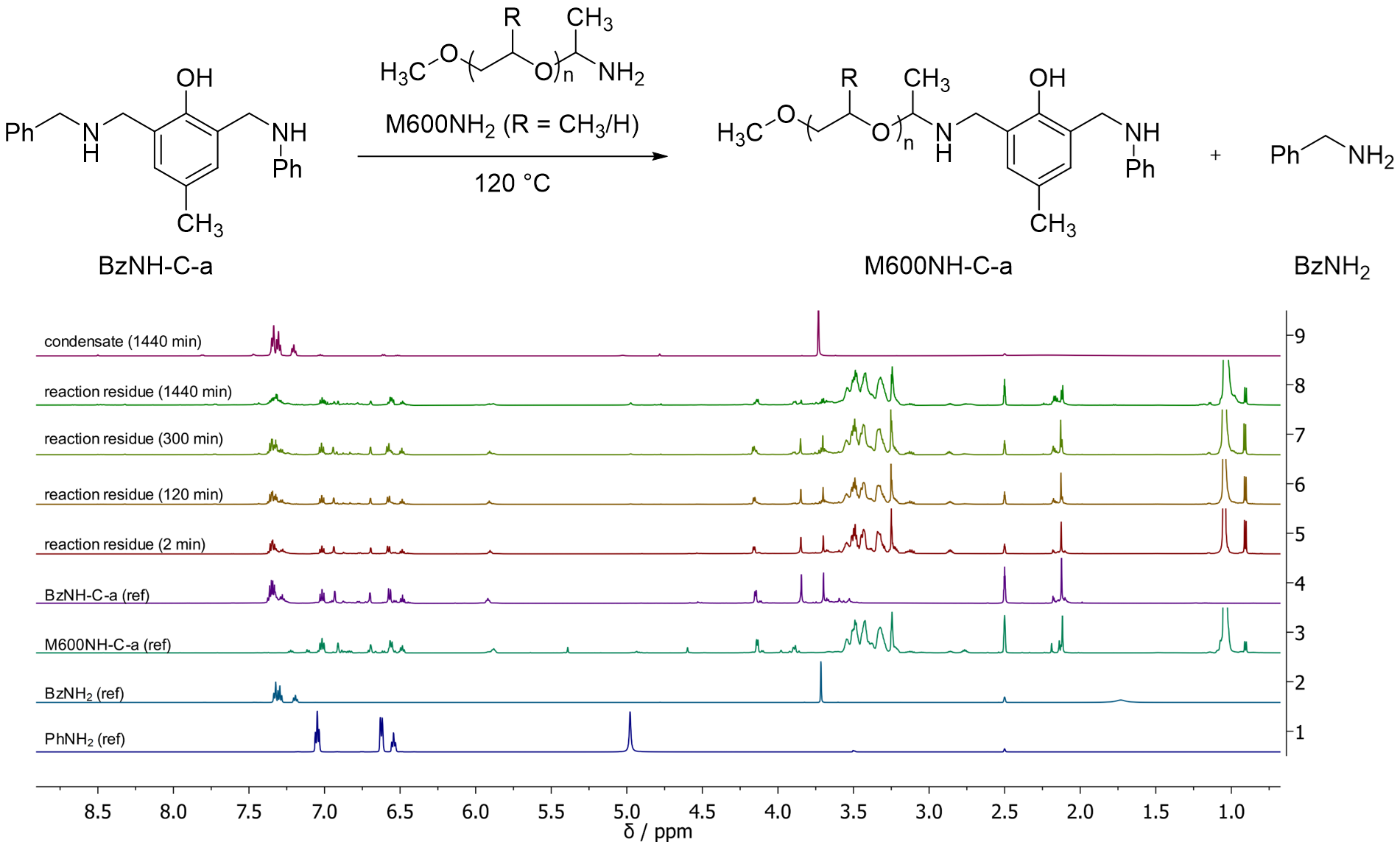
**

**Figure S16**: ^1^H-NMR (600 MHz, DMSO-d_6_, 296 K): Bulk reaction products (120 °C) of BzNH-C‑a with polyetheramine M600NH_2_, condensate above reaction mixture, as well as reference spectra of aniline PhNH_2_, benzylamine BzNH_2_, BzNH-C-a, M600NH-C-a.


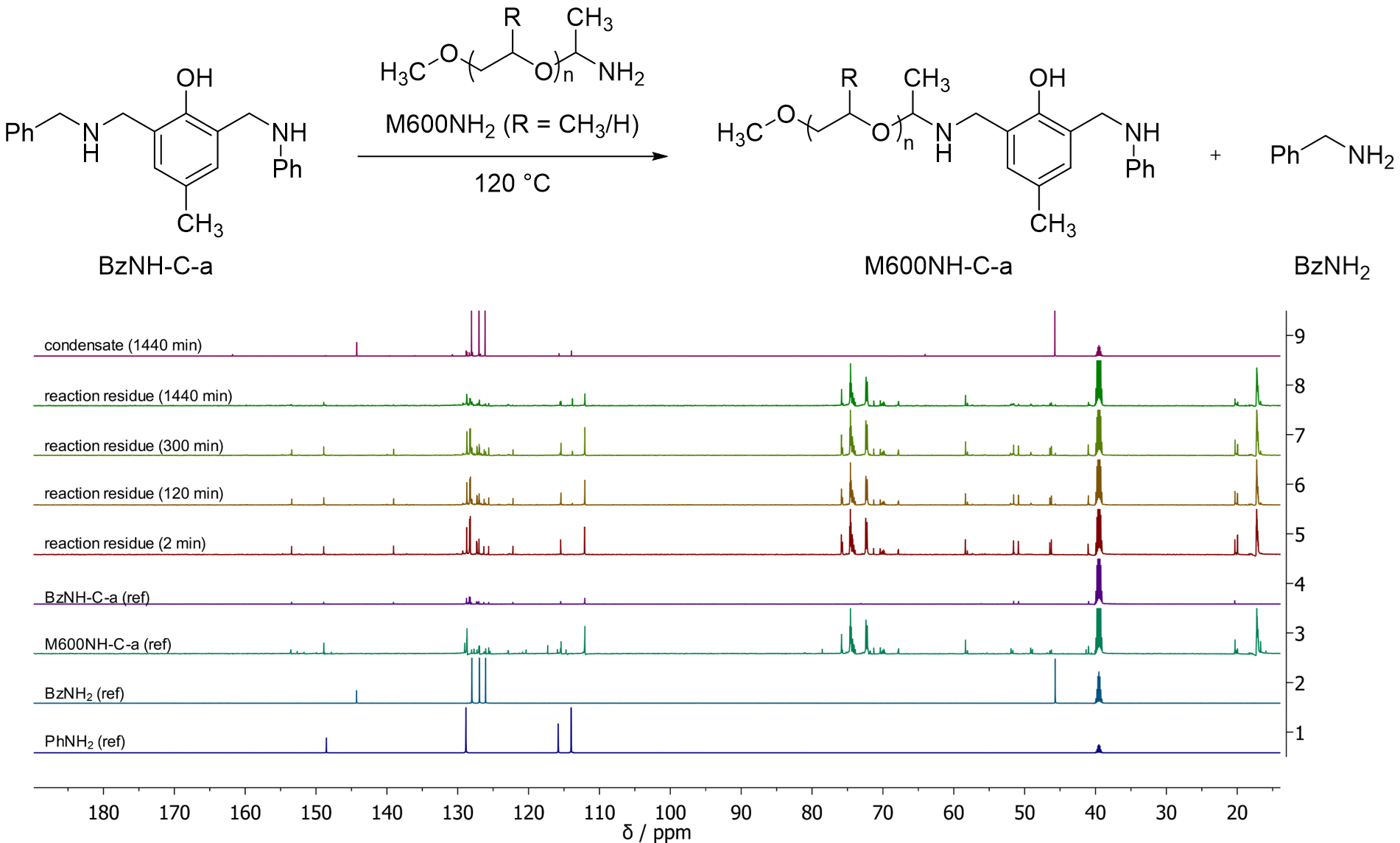


**Figure S17**: ^13^C{^1^H}-NMR (151 MHz, DMSO-d_6_, 296 K): Bulk reaction products (120 °C) of BzNH-C‑a with polyetheramine M600NH_2_, condensate above reaction mixture, as well as reference spectra of aniline PhNH_2_, benzylamine BzNH_2_, BzNH-C-a, M600NH-C-a.


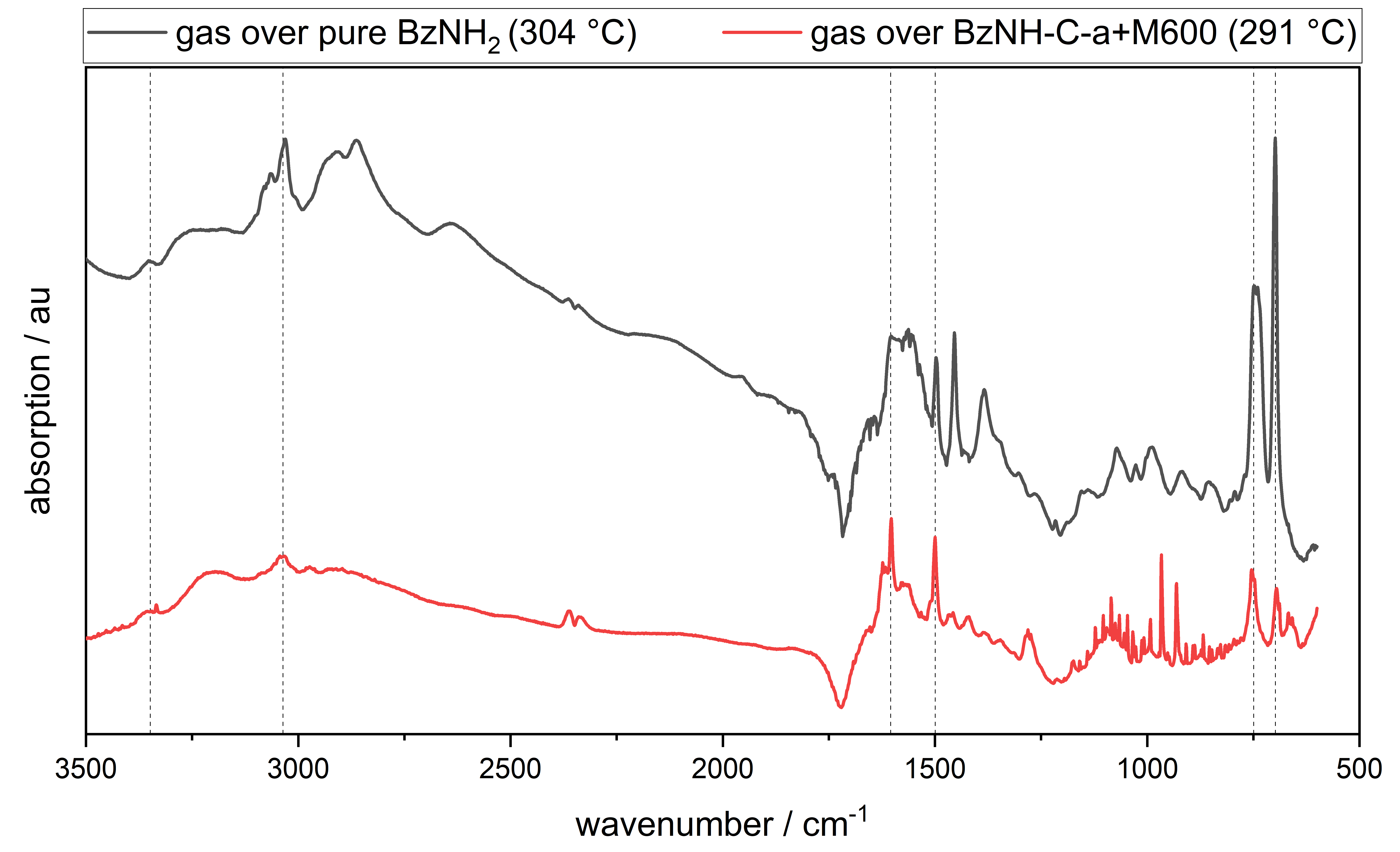


**Figure S18**: IR spectra of gas phase during TGA-IR measurement (35–550 °C, 10 K/min) of equimolar mixture of BzNH-C-a and M600 and pure BzNH_2_ as reference.





**Figure S19**: Gel contents of BA-a/ED600 polymers (BZ/amine) = (1:0, 1:0.5, 1:0.25, 1:0.125, neat BA-a) polymerized at different conditions with increasing polymerization temperatures for 2 h at each temperature: a) 120 °C; b) 120 °C, 150 °C; c) 120 °C, 150 °C, 180 °C; d) 180 °C, 200 °C (manufacturer protocol for neat BA‑a), heated in DMSO or solutions in DMSO (1 mol∙l^-1^) of benzylamine (BzNH_2_), aniline (PhNH_2_), dibenzylamine (Bz_2_NH), diphenylamine (Ph_2_NH), *N*,*N*-dimethylbenzylamine (Me_2_NBz) or *para*-cresol (C).

# 3. References

[29] L. Pursche, A. Wolf, T. Urbaniak, K. Koschek, *Front. Soft. Matter* **2023**, *3*, 1197868.

[35] T. Takeichi, K. Nakamura, T. Agag, H. Muto, *Des. Monomers Polym*. **2004**, *7*, 727.

# 4. Author Contributions

A.W. was responsible as lead for planning and execution of experimental work except stress-relaxation measurements, including evaluation of experimental data, prepared all figures and schemes, wrote the script. L.P. was responsible for planning, execution and evaluation of stress-relaxation measurements. L.B. and K.K. guided the research. K.K. and L.B edited the script. K.K. was responsible for the project supervision and funding.
